# Rare earth separations by selective borate crystallization

**DOI:** 10.1038/ncomms14438

**Published:** 2017-03-14

**Authors:** Xuemiao Yin, Yaxing Wang, Xiaojing Bai, Yumin Wang, Lanhua Chen, Chengliang Xiao, Juan Diwu, Shiyu Du, Zhifang Chai, Thomas E. Albrecht-Schmitt, Shuao Wang

**Affiliations:** 1 School for Radiological and Interdisciplinary Sciences (RAD-X), Soochow University and Collaborative Innovation Center of Radiation Medicine of Jiangsu Higher Education Institutions, 199 Ren'ai Road, Suzhou 215123, China; 2 Engineering Laboratory of Specialty Fibers and Nuclear Energy Materials, Ningbo Institute of Materials Technology and Engineering, Chinese Academy of Sciences, Ningbo, Zhejiang 315201, China; 3 Department of Chemistry and Biochemistry, Florida State University, 95 Chieftain Way, Tallahassee, Florida 32306, USA

## Abstract

Lanthanides possess similar chemical properties rendering their separation from one another a challenge of fundamental chemical and global importance given their incorporation into many advanced technologies. New separation strategies combining green chemistry with low cost and high efficiency remain highly desirable. We demonstrate that the subtle bonding differences among trivalent lanthanides can be amplified during the crystallization of borates, providing chemical recognition of specific lanthanides that originates from Ln^3+^ coordination alterations, borate polymerization diversity and soft ligand coordination selectivity. Six distinct phases are obtained under identical reaction conditions across lanthanide series, further leading to an efficient and cost-effective separation strategy via selective crystallization. As proof of concept, Nd/Sm and Nd/Dy are used as binary models to demonstrate solid/aqueous and solid/solid separation processes. Controlling the reaction kinetics gives rise to enhanced separation efficiency of Nd/Sm system and a one-step quantitative separation of Nd/Dy with the aid of selective density-based flotation.

## Abstract (web summary)

Trivalent lanthanides possess similar chemical properties, making their separation from one another challenging. Here, Wang and colleagues demonstrate that their subtle chemical differences can be greatly amplified during borate crystallization, leading to a low cost and highly efficient separation strategy.

Lanthanide (Ln) elements are a group of strategic resources and play an important role in the development of advanced materials, such as phosphors, permanent magnets, catalytic converters, lasers and batteries^1^,^2^,^3^. Ln^3+^ ions are dominant in solution and the solid state in most systems, and the valence 4*f* orbitals have minor to non-existent involvement in bonding^4^,^5^,^6^. The lanthanide contraction results in only a 0.15 Å decrease in ionic radii across the series and the average difference between neighbouring elements is only 0.01 Å (ref. 4). Thus, the combination of largely ionic bonding and lack of redox activity in aqueous media for most lanthanides results in chemical similarities between these elements that vexed researchers for many years. This ostensibly immutable chemistry inhibits both separations of these elements from one another and misidentification of elements especially neighbouring lanthanides in a number of instances^7^. Typically, lanthanide compounds form isostructural compounds; although a break from one family of compound to another often occurs at gadolinium^8^,^9^,^10^. This feature can also be treated as advantageous because it allows for the preparation of solid solutions with ratios of lanthanides that optimize desired physical properties^2^. One of the natural consequences of this phenomenon is that the 14 lanthanides along with lanthanum and yttrium often coexist in a variety of minerals, such as bastnasite LnFCO_3_ and monazite LnPO_4_ (refs 4, 7).

Developing separation methods for the *f*-block was one of the primary goals of the Manhattan Project. Spedding *et al*.^11^,^12^,^13^ at Ames Lab, used a series of ion-exchange resins to efficiently separate lanthanides. Rapid chemical separation of lanthanides and actinides using ion exchange resins with controlled parameters developed by Hoffman and Choppin *et al*. greatly promotes the investigations on the transplutonium elements and lanthanide fission products^14^,^15^,^16^. The LN and RE resins developed by Eichorm Co. have been commercially available for separations of lanthanides and actinides from 1990s (ref. 17). Despite these developments, challenges in scale up, continuous operation and resin regeneration/degradation still remain. In particular, biphasic solvent extraction methods offer solutions to many of these issues, and this technique was adopted on an industrial scale decades ago^18^. The largest concern associated with solvent extraction is the large amount of liquid waste generated. This waste is contaminated with uranium and thorium, in particular, because these elements are typical present in lanthanide minerals. Therefore, new separation strategies that are more environmentally benign are still being sought after^18^,^19^,^20^,^21^.

One of the earliest separation methods was fractional crystallization, which makes use of solubility differences between precipitates^7^. This technique was used to identify many elements in the periodic table before spectroscopic techniques became available in the early twentieth century, and in fact, was used to discover nuclear fission^22^. When used in lanthanide partitioning, its driving force originates primarily from the ionic radii differences, and hence low separation efficiency. With the recent advances in the *f*-element coordination chemistry, several strategies have emerged that can potentially enhance the separation efficiencies. A recent example shows an effective and simple method that can separate Nd/Dy based on the difference in the equilibrium constants for a coordination complex with a tripodal nitroxide ligand, where the separation factor reaches as high as 359 (ref. 23). Bu *et al*. utilized crystallization of two types of metal-organic frameworks to amplify the difference between early and late lanthanides giving rise to potential separations of two lanthanide ions with a large ionic radii differences^24^. However, the separation of lanthanides that are close in atomic numbers (for example, Nd/Sm) is still challenging and requires further enhancement of the differences among lanthanides in the solid state. To solve this issue, cooperative supramolecular coordination can potentially be utilized, and examples exist where lanthanide ions can be selectively recognized by rigid, tridentate ligands through fine-tuning kinetic and thermodynamic processes^25^,^26^,^27^,^28^,^29^.

Borates represent the most complex family of oxoanion compounds by virtue of boron's ability to occur within both BO_3_ triangles and BO_4_ tetrahedra. The linking of these units leads to almost limitless arrays of Fundamental Building Blocks (FBB)^30^,^31^. The polymerization of borates in aqueous systems is extremely sensitive to many external factors such as temperature, pH and concentrations and so ons^32^. When borates are polymerized around metal cations, the resultant polyborates display amplification of bonding differences between metal ions^33^. In short, grossly different compounds can be synthesized from neighbouring elements under the same reaction conditions^33^,^34^. Hence, this is an example of purely inorganic chemical recognition that is akin to cooperative supramolecular interactions. In the case of actinide borates, three different products with distinct crystal structures form with plutonium(III), americium(III) and curium(III) under the same reaction conditions; none of these products have trivalent lanthanide isotopic structures^34^. The lanthanide borate series were reported to occur in three different groups structure types that are not observed with trivalent actinides^34^. These fundamental studies provide hints that lanthanide separations might be achievable in a simple inorganic system if kinetic control of product formation can be achieved. In this report, we present substantial progress along this path by further amplifying the subtle chemical differences among trivalent lanthanides in the solid state during the crystallization of borates in molten boric acid. This observation leads to a lanthanide separation strategy that combines environmental friendliness, low cost and high efficiency.

## Results

### Periodic trend for the unary lanthanide borate system

**Figure 1:**
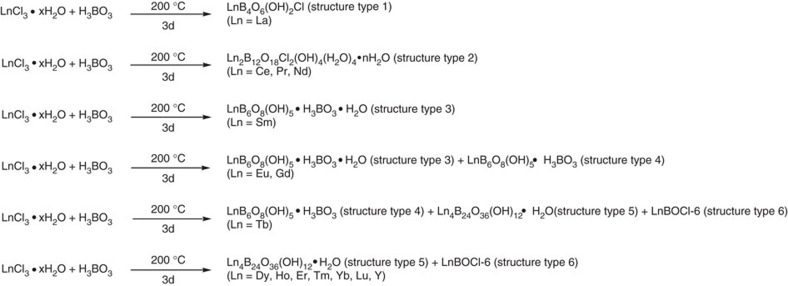
The periodic trend for the unary lanthanide borates from molten boric acid flux reactions. Chemical equations of boric acid flux reactions with six different groups of lanthanides showing the periodic trend for the formation of lanthanide borates.

When LnCl_3_˙xH_2_O reacts with large excess of molten boric acid at 200 °C for 3 days, six distinct phases across the lanthanide series were observed as shown in Fig. 1, establishing a unique periodic trend no longer showing chemical uniformity, clearly demonstrating the fine recognition capability in this system. Each reaction was reproducibly repeated for at least 10 runs and the products were examined and identified using a combination of single crystal X-ray diffraction, powder X-ray diffraction (PXRD, Supplementary Figs 1–14), and energy dispersive X-ray spectroscopy (EDS, Supplementary Figs 30–43). As shown in Fig. 1, the La reaction results in the formation of LaB_4_O_6_(OH)_2_Cl (LaBOCl-1, structure type 1) as a pure product^34^. By contrast, Ce, Pr and Nd reactions afford a different product of Ln_2_B_12_O_18_Cl_2_(OH)_4_(H_2_O)_4_˙nH_2_O (LnBOCl-2 as structure type 2, Ln=Ce, Pr, Nd), indicating Ce is the first transition point of the series. The pure Sm product, SmB_6_O_8_(OH)_5_˙H_3_BO_3_˙H_2_O (SmBOCl-3, structure type 3), represents the second transition, whose structure type can also be adopted by Eu and Gd (EuBOCl-3 and GdBOCl-3), but they produce additional phases of LnB_6_O_8_(OH)_5_˙H_3_BO_3_ (Ln=Eu as EuBOCl-4 and Gd as GdBOCl-4, structure type 4). Therefore, Eu can be considered as the third transition. The structure type 4 is also applicable to Tb (TbBOCl-4), which can be viewed as the fourth transition and contains two additional phases (Tb_4_B_24_O_36_(OH)_12_˙H_2_O as TbBOCl-5, structure type 5 and TbBOCl-6, structure type 6, whose formula is currently unknown). Starting from Dy as the last transition, all late lanthanides can only adopt the structure types 5 and 6. The same reactions are also conducted on the lanthanide analogues Sc and Y, and the results show that Sc does not form a crystalline product while Y forms solid products in structure types 5 and 6 (Supplementary Fig. 15), consistent with the periodic trend based on ionic radius.

One of the well-known lanthanide grouping phenomena is the tetrad effect, where the 15 lanthanides can be evenly sorted into four different groups (first group: La-Ce-Pr-Nd; second group: (Pm)-Sm-Eu-Gd; third group: Gd-Tb-Dy-Ho; fourth group: Er-Tm-Yb-Lu) to initially describe the M-shaped or W-shaped lanthanide distribution patterns in the solvent extraction system^35^,^36^,^37^. This effect originates from the variations of interelectronic repulsion for the ground states across the lanthanide series. Although the existence of the tetrad effect in natural media was a matter of debate^37^, it has been observed in lanthanide distribution patterns in seawater and many minerals^35^,^36^. In comparison, our observations on the periodic trend of the structure type evolution significantly deviate from the tetrad effect, where lanthanides are far from being evenly grouped based on the products obtained in the boric acid flux reaction.

### Structure and characterizations

**Figure 2:**
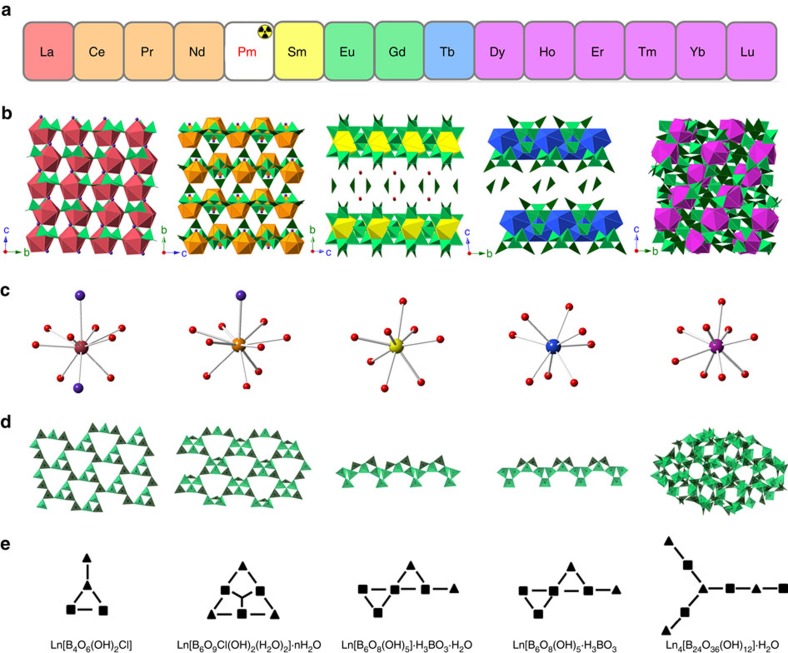
Periodic trend for the formation of lanthanide borates and crystal structures of five different structure types. (**a**) Periodic trend of lanthanides for the crystallization products; (**b**) depiction of the crystal structures; (**c**) Ln^3+^ coordination geometries; (**d**) borate networks; (**e**) borate FBB symbols. The lanthanide centres are shown as magenta, orange, yellow, blue or purple polyhedra/spheres, chlorine as mauve spheres, oxygen as red spheres, BO_4_ as light green tetrahedra and BO_3_ as dark green triangles.

We propose the observed discontinuity (Fig. 2a) is attributed to a combination of Ln^3+^ coordination variation, borate polymerization diversity (that is, metal-centre-reorganization leading to different borate FBBs in the crystallization products), and soft ligand coordination selectivity (that is, additional soft Cl donor would preferentially bind to early lanthanides, which are less polarizing than late ones), which is clearly revealed by the single crystal X-ray diffraction analysis. The structure type 1 contains 10-coordinate Ln^3+^ ions in capped triangular cupola geometry with *C*3*v* symmetry. Five BO_3_ triangles, six BO_4_ tetrahedra, a bridging Cl^−^ and a terminal Cl^−^ are found in the first coordination sphere of Ln^3+^ ions (Fig. 2c). The FBB of the polyborate network is a tetraborate unit consisting of two BO_3_ triangles and two BO_4_ tetrahedra as shown in Fig. 2e. In comparison, the structure type 2 contains 10-coordinate Ln^3+^ ions also in capped triangular cupola geometry (Fig. 2c). However, the constituent in the first coordination sphere is different containing five BO_3_ triangles, five BO_4_ tetrahedra, two H_2_O molecules and a terminal Cl^−^, further leading to a different polyborate anion with a hexaborate FBB consisting of three BO_3_ triangles and three BO_4_ tetrahedra (Fig. 2e).

Although Cl^−^ is used in the starting materials, it does not participate in bonding with middle and late lanthanides starting from Sm owing to the soft ligand coordination selectivity, leading to structure types 3 and 4 with prominent deviations from the first two structure types. LnO_8_ polyhedral with coordination geometry of slightly distorted square antiprism and another type of hexaborate FBB containing two BO_3_ triangles and four BO_4_ tetrahedra are found in both structures (Fig. 2e), forming layered structures (Fig. 2b), which are quite similar with the recently reported trivalent californium borate Cf[B_6_O_8_(OH)_5_]^38^. However, as can be probed by the crystal structure depiction and the molecular formula, these two structures also exhibit clear deviation from each other. In fact, the structure type 3 crystallizes in the space group of *C*2*/m*, whereas structure type 4 is in *P*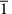, resulting in deviation on the layer stacking style and moieties filled in the interlayer space as shown in Fig. 2b. By contrast, the structure type 5 adopted by late lanthanides is much more complex than the first four structures. It contains two crystallographically independent LnO_9_ polyhedra adopting the coordination geometry of distorted tricapped trigonal prism, where six BO_3_ triangles and six BO_4_ tetrahedra are found in the first coordination sphere of Ln^3+^ (Fig. 2c). A borate FBB containing four BO_3_ triangles and four BO_4_ tetrahedra is observed, forming a previously unknown octaborate unit (Fig. 2e). These FBBs are further polymerized into a dense 3D network (Fig. 2d).

### Binary lanthanide separation models

Given a unique periodic trend with significantly amplified differences is discovered, a series of binary lanthanide crystallization experiments were conducted to initially investigate its application on separations, especially between lanthanides forming different structures. Twelve combinations (La/Ce, Ce/Nd, Ce/Eu, Ce/Lu, Nd/Sm, Nd/Dy, Sm/Eu, Eu/Lu, Gd/Tb, Tb/Dy, Dy/Ho and Dy/Lu) were selected by reacting a mixture of two different LnCl_3_˙xH_2_O in 1:1 molar ratio with excess of boric acid at 200 °C for 3 days. Among these, combinations of Nd/Sm, Nd/Dy, Ce/Eu, Ce/Lu and Eu/Lu represent the cases of two lanthanides from two different groups in the observed periodic trend while combinations of Ce/Nd and Dy/Lu are examples for two lanthanides from the same group. The mixed crystallization products were characterized initially by the PXRD to indentify the structure type (Supplementary Figs 16–27).

**Figure 3:**
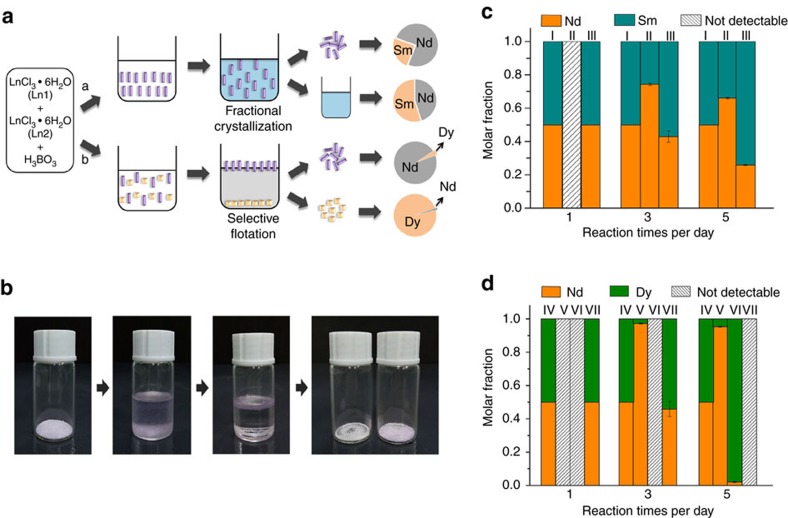
Binary lanthanide separation models and results. (**a**) Depiction of lanthanide separation strategies (top: solid/aqueous separation for Nd/Sm; bottom: solid/solid separation for Nd/Dy) based on the enhanced difference for crystallization products; (**b**) photos showing the process of selective flotation in the Nd/Dy solid/solid separation experiment; (**c**) separation results of Nd/Sm crystallization experiment (I, II and III columns represent the molar distribution of Nd/Sm in the starting material, NdBOCl-2 crystals and wash solutions, receptively); (**d**) separation results of Nd/Dy crystallization experiment (IV, V, VI and VII columns represent the molar distribution of Nd/Dy in the starting material, NdBOCl-2 crystals, DyBOCl-5/DyBOCl-6 crystals and wash solutions, respectively).

The combination of Nd/Sm is an important and interesting one to investigate given that they only differ by two atomic numbers but adopt substantially different structure types. The PXRD results reveal that the mixed Nd/Sm reaction only afford crystalline products in structure type of NdBOCl-2. That is, the presence of Nd in the reaction prevents Sm from formation of its own preferential product; Sm is therefore not able to efficiently incorporate into the crystals at the same magnitude of Nd, giving rise to a potential selective crystallization mechanism. Indeed, as initially examined by EDS analysis, the measured average atomic ratio of Nd:Sm for 10 randomly selected crystals is 2.7:1, whereas Sm mostly remains in the water-soluble portion as unreacted SmCl_3_˙xH_2_O. Therefore, the separation of Nd and Sm can be simply achieved by washing the solid using hot water followed by solid/aqueous separation as shown in Fig. 3a. In order to precisely determine the separation factor, the crystalline solids were further dissolved in nitric acid and the atomic ratio was quantitatively analysed using inductively coupled plasma mass spectrometry (ICP-MS) along with the wash solution sample. The measured Nd/Sm separation factor is 4.03±0.26, which is already at the same level with the most solvent extraction processes (ranging from 2.2 to 4.86) without further optimizations^18^. It should be noted that for traditional fractional crystallization based on phosphates without recognition capability, the separation factor was reported to be 1.18 (ref. 39).

The 3 days reactions for combinations of Ce/Eu, Nd/Dy, Eu/Lu and Ce/Lu are similar with that of Nd/Sm, but with larger separation factors as shown in Supplementary Fig. 53. Impressively for the case of Ce/Lu, Lu was not even detectable in the solid product by EDS analysis and determined to be in trace amount by ICP-MS analyses, resulting in a high separation factor of 716.85±14.89, which can also be substantially increased by elongating the reaction time (see discussion part; for comparison, the separation factor determined for the industrial M^III^-HCl-HDEHP (HDEHP=di-(2-ethylhexyl)phosphoric acid) extraction method is 199 (ref. 18)). By contrast, the combinations of two lanthanides forming the same structure type exhibit lower separation factors compared with those combinations forming different structure types. The difference between separation factors of 3.11±0.14 for Ce/Nd and 4.03±0.26 for Nd/Sm (both with atomic number difference of 2) seems small but in fact significant, given those values in the traditional fractional crystallization based on phosphates are both extremely small and similar at 1.38 and 1.18, respectively. For the case of Dy/Lu with the atomic number difference of 5, the separation factor is only 1.59±0.12, contrasting sharply with the case of Nd/Sm. The low-separation factor of Dy/Lu originates mainly from the Ln^3+^ ionic radii difference, which highly resembles the case of traditional fractional crystallization strategy with extremely low-separation efficiencies. Consequently, all these results unambiguously demonstrate the power for separation efficiency enhancement originating from the amplified structural difference on the crystallization products.

The combinations of two lanthanides that are adjacent but form different products (for example, La/Ce, Sm/Eu, Gd/Tb and Tb/Dy) are also investigated while the combination of Dy/Ho represents the adjacent lanthanides forming the same product. For the case of La/Ce, the products consist of mixed solid products in both structure types 1 and 2 shown by PXRD data (Supplementary Fig. 16). These solids are not distinguishable by morphology, leading to a relatively low-separation factor of 1.43±0.02 based on the solid/liquid separation model. However, this result does not exclude the possibility of enrichment of La/Ce in their own preferential phases. The combinations of Sm/Eu, Gd/Tb and Tb/Dy all exhibit low separation factors below 2 (Supplementary Fig. 53), but showing small improvements over the case of Dy/Ho. This is not surprising because these combinations of two lanthanides can form solid products overlapped with each other (for example, Tb/Dy both form structure types 5 and 6).

### Enhanced separation by controlling the reaction kinetics

If two lanthanide ions are incorporated in the crystalline phase in a fixed molar ratio, the separation factor would greatly depend on the yield of the crystalline phase. Therefore, a higher yield of solids would result in a higher separation factor. In order to confirm this hypothesis, a series of time-dependent binary lanthanide crystallization reactions (1, 3 and 5 days) were performed and analysed. Taking the Nd/Sm reaction as an example, the results show that no solid formed in the 1 day reaction, and therefore separation cannot be achieved. Formation of the same NdBOCl-2 structure was observed for the rest of reaction times (Supplementary Figs 20 and 28; EDS analysis is shown in Supplementary Figs 47 and 51). ICP-MS analysis reveals that the yield of the crystalline phase for Nd is low at 26% while 74% of Nd still remains in the water-soluble portion for the 3 days reaction. As expected, the elongation of reaction time to 5 days leads to an increase of crystallization yield to 77% for Nd. However, the molar ratio of Nd/Sm in the solid state is lowered from 3.06 to 1.88, resulting in a corresponding sacrifice of separation efficiency; despite that the separation factor still increases to 5.32±0.18 owing to the increase of crystallization yield (Table 1). Clearly, the differential crystallization kinetics between two Nd/Sm are induced by the intrinsic preference of solid phase formation, which differs substantially from the traditional fractional crystallization strategy, where the subtle solubility differences are the only thermodynamic driving force.

The crystallization kinetics of the Nd/Dy reaction represents a completely different story and gives rise to a significant optimization of separation efficiency. Similarly, 1 day reaction does not yield any crystalline products, whereas 3 days reaction affords crystals of NdBOCl-2 dominated with Nd while Dy almost entirely remains in the water-soluble portion similar to the case of Ce/Lu. However, 5 days reaction results in the formation of additional crystalline products of DyBOCl-5 and DyBOCl-6 phases (Supplementary Figs 21 and 29). More importantly, as shown in Table 2, the ICP analysis on the wash solution samples indicate that both Nd and Dy are almost completely crystallized into their preferential solid phases while the incorporation rates in each other's phase is quite low shown by EDS analysis on the crystals (Supplementary Figs 48 and 52), resulting in a potential one-step quantitative separation with a recovery rate close to 100% (Fig. 3d). This observation contrasts sharply with the concept of the solid solution of lanthanides that the atomic ratio of two lanthanides in the crystallization solid product can be simply predicted by the atomic ratio of two lanthanides in the starting material of a crystallization reaction. To separate the crystals of NdBOCl-2 from DyBOCl-5 and DyBOCl-6, the selective flotation method can be utilized because the theoretic density for the relatively porous structure of NdBOCl-2 (2.70 g cm^−3^) is significantly lower than that of the dense structures of both DyBOCl-5 (3.38 g cm^−3^) and DyBOCl-6 (although the precise density for this phase is not available, a higher density than that of DyBOCl-5 was predicted by analysing the incomplete electron density map). Indeed, as shown in Fig. 3b, when soaking the mixed products in bromoform (density: 2.89 g cm^−3^), crystals of NdBOCl-2 rapidly move to the top of the solution while crystals of DyBOCl-5 and DyBOCl-6 remain in the bottom, leading to a complete separation of Nd/Dy phases as demonstrated by the PXRD measurements. The ICP analysis on the solutions of dissolved solid samples from bottom and supernatant products gives a promising separation factor of 986.33±57.15 between Nd/Dy (Table 2). By contrast, the separation factor for the single stage solvent extraction process using the extractant HDEDP is in the range of 40–250 (ref. 18). Although bromoform used here cannot be truly considered as a green solvent, the interaction between bromoform and borate crystals are not observed. Therefore, the bromoform can be fully and facilely recycled by filtration or centrifugation. In addition, the relatively low cost can be expected in general for binary lanthanide separations given the major separation reagent and reaction medium boric acid is quite cheap (ca. 1 dollar per gram).

## Discussion

To further elucidate the observed discontinuity from the theoretical view, first-principles computations are carried out by the density functional theory method^40^. The reaction energies for the reactions to produce structure type 2, defined by the energy difference between products and reactants, for some lanthanide (La, Ce, Sm, Gd, Td and Lu) are predicted; the partial density of states (PDOS) for the exemplary lanthanide (Ce, Sm and Gd) borates are plotted as well. The computational results and analyses are provided in Supplementary Fig. 54. It is found that the reaction energy difference may be amplified from in the lanthanide borate despite of the subtlety in their radius. The analysis on PDOS also indicates that the behaviours of inner orbitals are influenced differently in different lanthanide borates, which may lead to apparent distinctions in ligand-centre coordination causing distinguishable borate phases. However, one should be aware that these calculations are based on the electronic structures of the lanthanide borate products on the microscopic scale.

It should be accentuated that the phases determined experimentally show diversity for different lanthanides and the products are strongly dependent on the reaction time (such as Nd/Dy) and the co-crystallized elements (such as Nd/Sm). This indicates the reactions for different lanthanides occur with drastically different kinetics and thus are unlikely to be solely thermodynamically driven. Namely, the kinetics of reaction, grain growth and diffusion and interfacial interaction of grain boundaries may all play significant roles in determining the crystallization products, which means some factors should be theoretically elucidated from the evolution of the microstructures in the mesoscale. In the application perspective, since the scheme of selective borate crystallization has been demonstrated to be effective for lanthanide separation in our study, the optimizations on the procedures and conditions are naturally expected to be able to further enhance the separation efficiency, and the work is now ongoing.

The foregoing results demonstrate that the subtle differences among trivalent lanthanides can be greatly amplified during selective borate crystallization in molten boric acid, breaking our basic understanding on the fundamental lanthanide chemistry by assuming their high chemical similarities. This observation further leads to a rare earth separation strategy with combined advantages of environmental friendliness, low cost and high efficiency. This strategy is substantially different with the conventional separation methods based on incremental variation of thermodynamic parameters with atomic numbers. We expect that an inorganic recognition system with improved crystallization kinetics following this idea should show real utilities in the near future.

## Methods

### Materials

All reagents were purchased from chemical reagent suppliers and used without further purification. Lanthanide chlorides including LaCl_3_·6H_2_O (99.99%), NdCl_3_·6H_2_O (99.99%), SmCl_3_·6H_2_O (99.99%), EuCl_3_·6H_2_O (99.99%), GdCl_3_·6H_2_O (99.99%), TbCl_3_·6H_2_O (99.99%), DyCl_3_·6H_2_O (99.99%), HoCl_3_·6H_2_O (99.99%), ErCl_3_·6H_2_O (99.99%), TmCl_3_·6H_2_O (99.99%), YbCl_3_·6H_2_O (99.99%) and LuCl_3_·6H_2_O (99.99%) were provided from Energy Chemical Reagent Co., Ltd. China. Others: CeCl_3_·7H_2_O (99%, Alfa Aesar Co.), PrCl_3_ (99.99%, Aladdin Co.), H_3_BO_3_ (≥99.5%, Chinasun Specialty Products co., Ltd.), Bromoform (CHBr_3,_ 97%, Alfa Aesar Co.) and Chloroform (CHCl_3_, ≥99.0%, Yonghua Chemical Technology Co., Jiangsu, China).

### Synthesis of lanthanide borates

Individual lanthanide chloride LnCl_3_˙xH_2_O (0.3 mmol) and boric acid (4.5 mmol) were charged into a PTEF-lined Parr 4749 autoclave with a 10 ml internal volume, then dissolved using 100 μl of deionized water. Subsequently, the samples were sealed and heated at 200 °C for 3 days followed by slow cooling to room temperature over a period of 2 days. The resulting products were washed extensively with boiling water to remove excess boric acid. The crystal products were then washed by ethanol and dried at room temperature. Six different phases of lanthanide borates were obtained across the lanthanide series under indentical reaction condition, as shown by PXRD from Supplementary Figs 1–14. Crystallographic information is listed in Supplementary Table 1, atomic coordinate and additional structural information are provided in the Supplementary Data 1. All these crystals were further characterized by EDS analysis (Supplementary Figs 30–43).

### Binary lanthanide separation by borate crystallization

Nd/Sm separation: NdCl_3_˙6H_2_O (0.3 mmol), SmCl_3_˙6H_2_O (0.3 mmol) and boric acid (4.5 mmol) were charged into a PTEF-lined Parr 4749 autoclave with a 10 ml internal volume, then dissolved using 100 μl of deionized water. The samples were sealed and heated at 200 °C for 3 days followed by slow cooling to room temperature over 2 days. The resulting products were washed extensively with boiling water to remove excess boric acid. The wash solutions were gathered in a volumetric flask and added to a constant volume with deionized water for further determination of the molar quantities of Nd and Sm. The products were then washed with ethanol and dried at room temperature. The mixed crystallization products were characterized by the PXRD to identify the structure type (Supplementary Fig. 20), and the molar ratio of Nd: Sm was initially examined by energy dispersive spectroscopy analysis (EDS) (Supplementary Fig. 47). In order to precisely determine the quantities of Nd and Sm in solids, the crystalline solids were further dissolved in concentrated nitric acid, and then diluted to 5% nitric acid solution before quantitatively analysed using ICP-MS, the wash solution samples were also analysed in order to determine the separation factor. Separation factors are calculated using the following equation


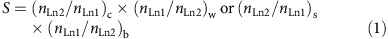


Crystallization yield are calculated as


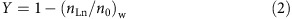


where *n*_Ln1_, *n*_Ln2_ is the molar mass two different lanthanides, *n*_0_ is the initial molar mass of certain lanthanide charged into the autoclave. c represents the case in the solid products, w represents the case in the wash solutions, s represents the case in the supernatant products after selective flotation and b represents the case in the bottom products after selective flotation. For Nd/Dy reaction, the molar mass in products is calculated by following


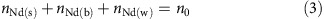



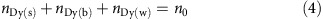



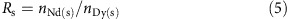



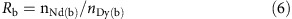


where the molar masses of *n*_Nd(w)_ and *n*_Dy(w)_ in wash solutions are in trace amount as determined by ICP analysis, *R*_s_ is the molar ratio of Nd/Dy in the supernatant products, and *R*_b_ is the molar ratio of Nd/Dy in bottom products determined by ICP analysis.

### Other binary lanthanide separations

The other eleven binary lanthanide separations of La/Ce, Ce/Nd, Ce/Eu, Ce/Lu, Nd/Dy, Sm/Eu, Eu/Lu, Gd/Tb, Tb/Dy, Dy/Ho and Dy/Lu were under the same procedure with Nd/Sm binary separation, the structure type was determined by PXRD, as shown in Supplementary Figs 16–19 and 21–27. The corresponding molar ratios of lanthanide elements are shown in Supplementary Figs 44–46 and 48–50.

### Enhanced separation by controlling reaction kinetics

Nd/Sm separation: The reaction conditions and processes are similar with the procedure above except that the reaction time was extended to 5 days. The PXRD show the mixed crystallization products contain only one structure type (NdBOCl-2) in products (for 5 days Supplementary Fig. 28), and the atomic ratio of Nd:Sm was initially examined by EDS analysis (Supplementary Fig. 51). The wash solution was also analysed using the ICP-MS to precisely determine the molar ratio in the crystalline solids. The separation factor and crystallization yields are listed in Table 1. One day reaction time was also tested, however, no crystallization products can be found. Nd/Dy separation: The reaction conditions and processes are similar with the procedure above except that the reaction time was extended to 5 days. The PXRD show the mixed crystallization products contain three distinct structure types (NdBOCl-2, DyBOCl-5 and DyBOCl-6, Supplementary Fig. 29), and the atomic ratio of Nd/Dy was initially examined by EDS analysis for the products in different structural types (Supplementary Fig. 52). The products were then separated by the selective flotation method using a mixed bromoform and chloroform solvent (V(CHBr_3_):V(CHCl_3_)=25:4). After this, the supernatant products and bottom products were separated and characterized by PXRD (Supplementary Fig. 29), and then dissolved in concentrated nitric acid to determine the separation factor.

### Characterizations

PXRD data were collected from 5 to 50° with a step of 0.02° and the time for data collection was 0.2–0.5 s on a Bruker D8 Advance diffractometer with Cu Kα radiation (*λ*=1.54056 Å). EDS data were collected using FEI Quanta 200FEG. The energy of the electron beam was 30 kV, and the spectrum acquisition time was 100 s. ICP-MS analysis of separation experiment was conducted using a Thermo Finnigan high-resolution magnetic sector Element 2 ICP-MS instrument. Inductively coupled plasma optical emission spectrometer (ICP-OES) analysis of separation was conducted using a Thermo Scientific ICAP 7400 instrument. Single crystal X-ray diffraction measurements were performed using a Bruker D8-Venture single crystal X-ray diffractometer equipped with a digital camera. The diffraction data were collected using a Turbo X-ray Source (Mo–Kα radiation, *λ*=0.71073 Å) adopting the direct-drive rotating anode technique and a CMOS detector under room temperature. The data frames were collected using the programme APEX2 and processed using the programme SAINT routine in APEX2. The structures were solved by the direct method and refined on F^2^ by full-matrix least-squares methods using SHELXTL-2014 programme.

### Computational details

The models for LnBOCl-2 with Ln=La, Ce, Sm, Gd, Tb and Lu are constructed according to the corresponding experimental crystal structures. Our first-principles density functional theory calculations are performed with the CASTEP codes^41^,^42^, which use a plane wave basis set for the valence electrons and ultrasoft pseudo-potential for the core electrons. The spin-polarized Perdew–Breke–Ernzerh^43^,^44^ functional under generalized gradient approximation^45^,^46^ is adopted for the exchange and correlation energy. The valence electron configurations for all atoms are chosen to be conventional, that is, H-1s^1^, B-2s^2^2p^1^, O-2s^2^2p^4^, Cl-3s^2^3p^5^ and La-5s^2^5p^6^5d^1^6s^2^, Ce-4f^1^5s^2^5p^6^5d^1^6s^2^, Sm-4f^6^5s^2^5p^6^6s^2^, Gd-4f^7^5s^2^5p^6^5d^1^6s^2^, Tb-4f^9^5s^2^5p^6^6s^2^, Lu-4f^14^5p^6^5d^1^6s^2^. Total energy changes in self-consistent calculations are finally reduced to less than 1 × 10^−5^ eV per atom, and Hellman–Feynman forces acting on atoms are converged to less than 0.03 eV/Å.

### Data availability

The X-ray crystallographic coordinates for structures reported in this work have been deposited at the Cambridge Crystallographic Data Centre (CCDC), under deposition numbers CCDC 1521491-1521495. These data can be obtained free of charge from CCDC via www.ccdc.cam.ac.uk/data_request/cif. All other data are either provided in the Article and its Supplementary Information or are available upon request.

## Additional information

**How to cite this article:** Yin, X. *et al*. Rare earth separations by selective borate crystallization. *Nat. Commun.* **8,** 14438 doi: 10.1038/ncomms14438 (2017).

**Publisher's note:** Springer Nature remains neutral with regard to jurisdictional claims in published maps and institutional affiliations.

## Supplementary Material

Supplementary Information Supplementary Figures and Supplementary Table

Supplementary Data 1 Combined crystallographic information files.

## Figures and Tables

**Table 1:** Result summary of Nd/Sm separation experiment.

**Reaction times**	**Element**	**The molar mass in reactants per mmol**	**The molar mass in products per mmol**	**The molar mass in wash solutions per mmol**	**Crystallization yield**	Separation factor*
3d	Nd	0.3±0.015	0.0496±0.0026	0.2204±0.0197	0.2653±0.0104	4.03±0.22
	Sm	0.3±0.015	0.0162±0.0011	0.2861±0.0082	0.0493±0.0011	
5d	Nd	0.3±0.015	0.2053±0.0024	0.0700±0.0020	0.7667±0.0361	5.32±0.18
	Sm	0.3±0.015	0.1090±0.0020	0.1963±0.0009	0.3457±0.0157	

^*^The separation factor is calculated using the solid/aqueous model.

**Table 2:** Result summary of Nd/Dy separation experiment.

**Reaction times**	**Element**	**The molar mass in reactants per mmol**	**The molar mass in NdBOCl-2 phase per mmol**	**The molar mass in LnBOCl-5 /LnBOCl-6 phase per mmol**	**The molar mass in wash solutions per mmol**	**Crystallization yield**	Separation factor*
3d	Nd	0.3±0.015	0.0412±0.0036	—	0.1954±0.0047	0.3487±0.0090	67.76±0.60
	Dy	0.3±0.015	0.0007±4E-5	—	0.2248±0.0151	0.2607±0.0045	
5d	Nd	0.3±0.015	0.2960±0.0034	0.0058±0.0003	5.6E-5±1.0E-5	0.9999±0.0500	986.33±57.15
	Dy	0.3±0.015	0.0147±0.0012	0.2871±0.0033	3.4E-5±0.6E-5	0.9999±0.0500	

^*^Only the structure of NdBOCl-2 was observed in the solid product in the 3d reaction, therefore, the separation factor is calculated using the solid/aqueous model. The separation factor is calculated using the solid/solid model for the 5d reaction.
