# Supplementary material for: Rare earth separations by selective borate crystallization
Source: Nat Commun. 2017 Mar 14;8:14438. doi: 10.1038/ncomms14438 (PMC5355876; doi:10.1038/ncomms14438)
Supplement: Supplementary Information — Supplementary Figures and Supplementary Table [file ncomms14438-s1.pdf]

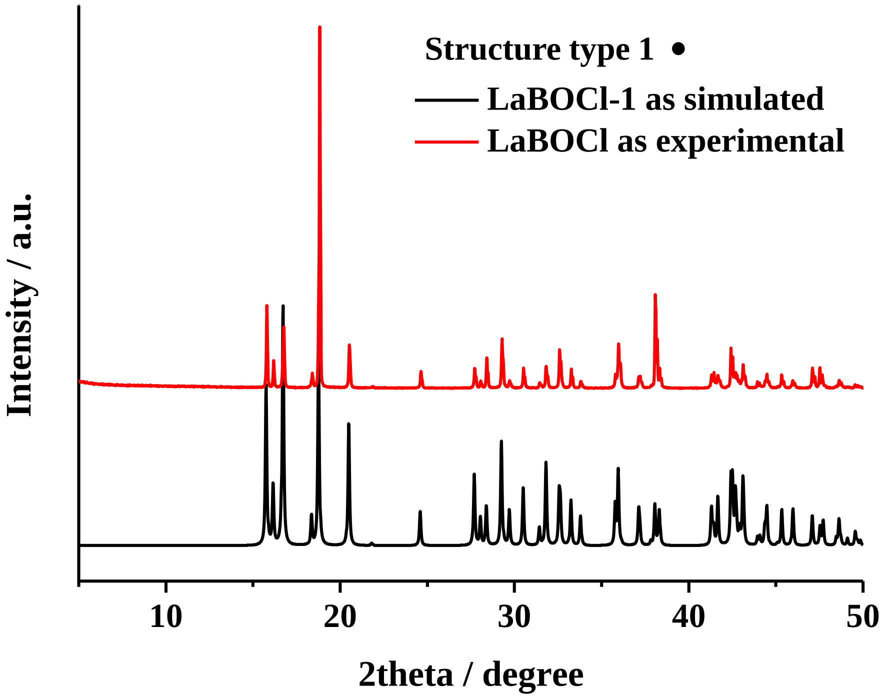

Supplementary Figure 1. Powder X-ray diffraction (PXRD) patterns of LaBOCl synthesized at 3 days

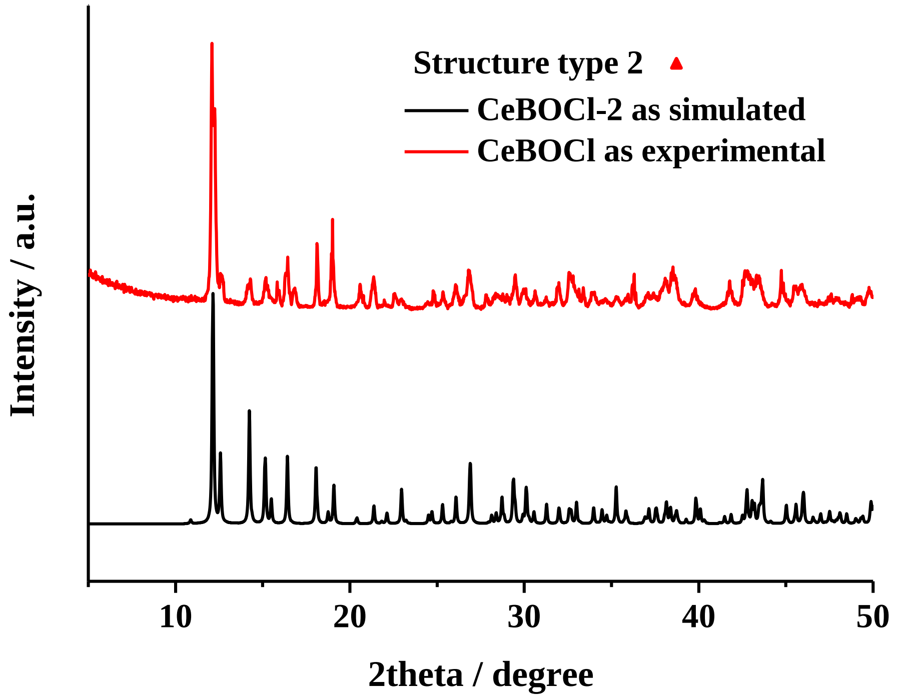

Supplementary Figure 2. Powder X-ray diffraction (PXRD) patterns of CeBOCl synthesized at 3 days

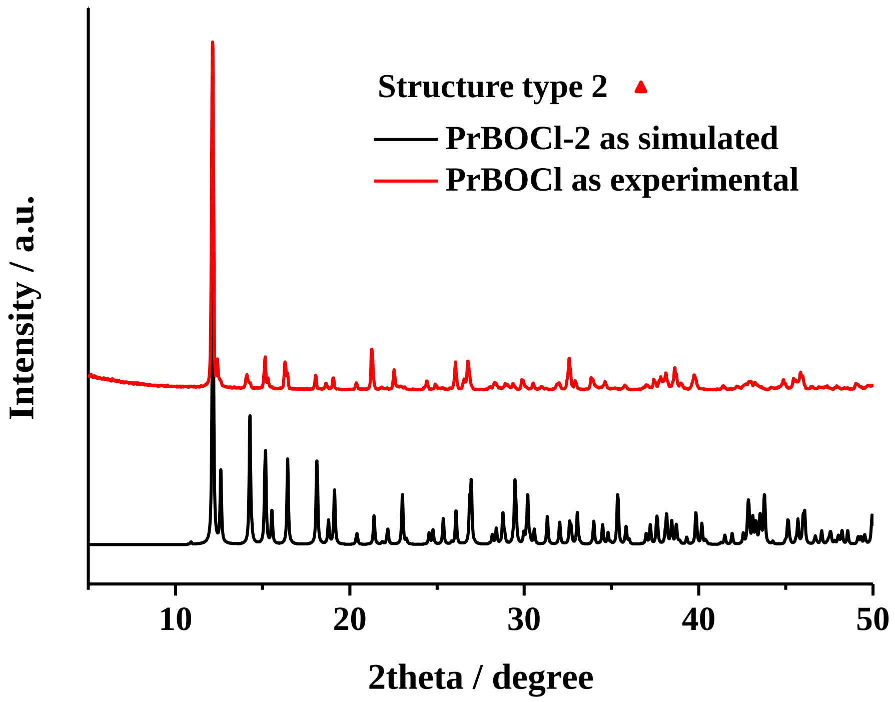

Supplementary Figure 3. Powder X-ray diffraction (PXRD) patterns of PrBOCl synthesized at 3 days

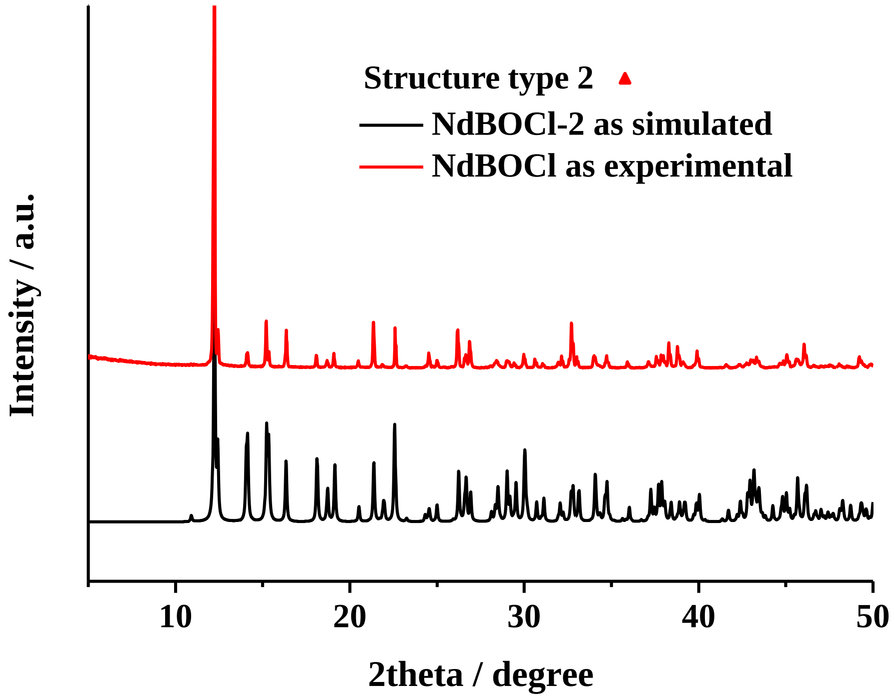

Supplementary Figure 4. Powder X-ray diffraction (PXRD) patterns of NdBOCl synthesized at 3 days

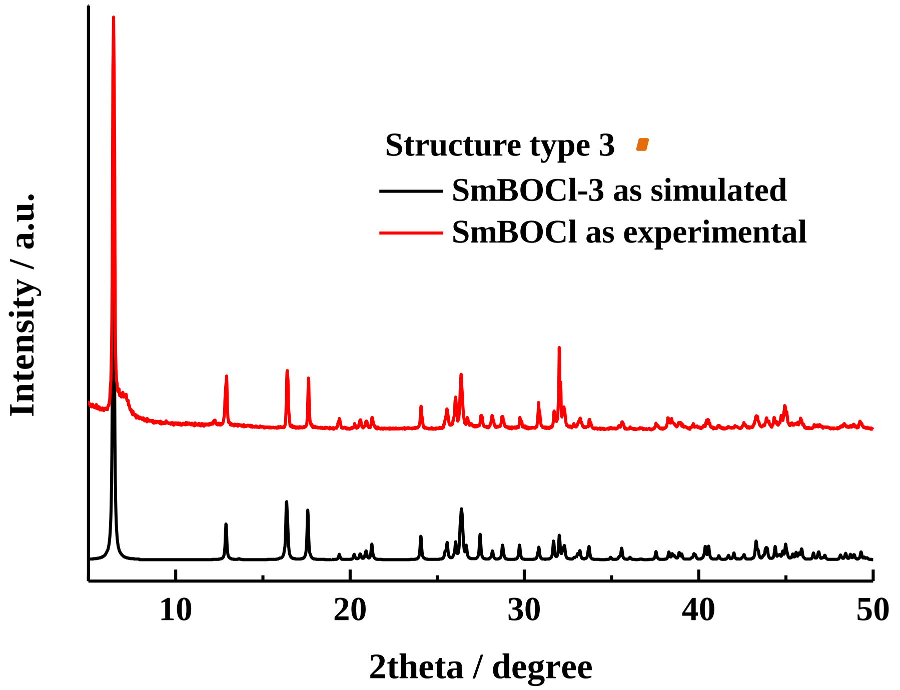

Supplementary Figure 5. Powder X-ray diffraction (PXRD) patterns of SmBOCl synthesized at 3 days

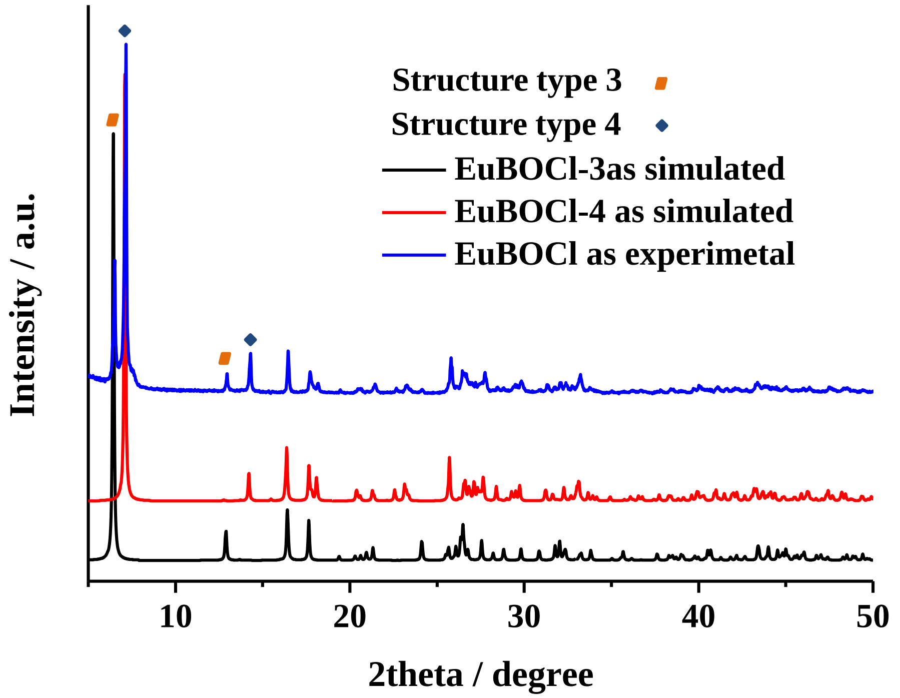

Supplementary Figure 6. Powder X-ray diffraction (PXRD) patterns of EuBOCl synthesized at 3 days

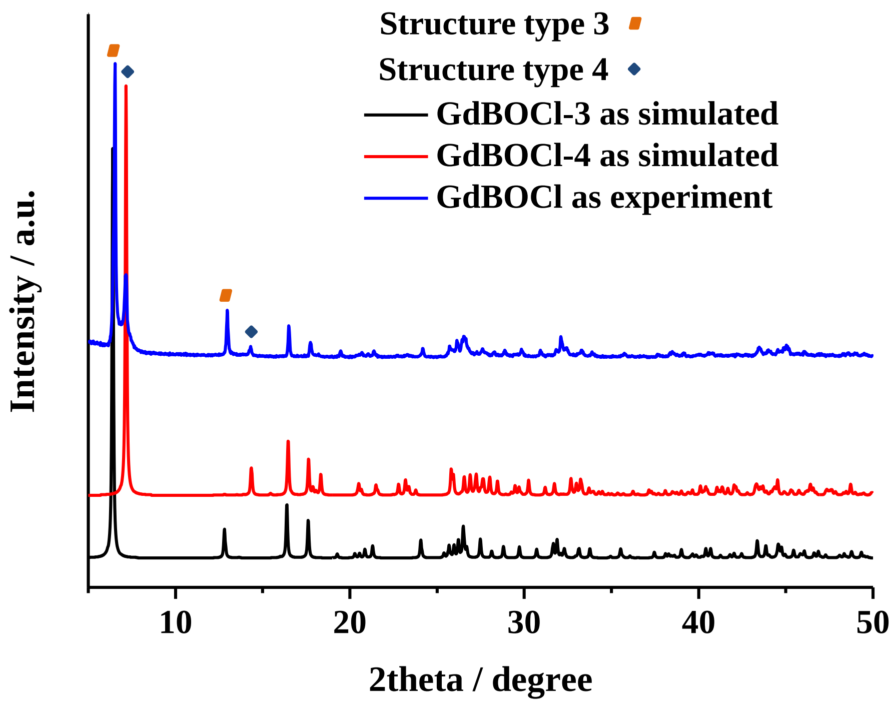

Supplementary Figure 7. Powder X-ray diffraction (PXRD) patterns of GdBO synthesized at 3 days

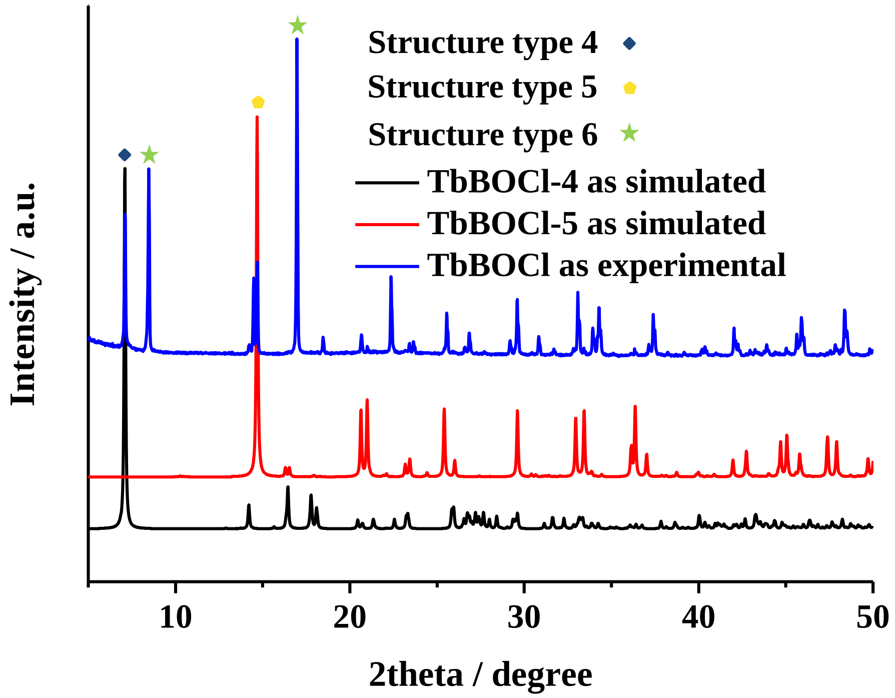

Supplementary Figure 8. Powder X-ray diffraction (PXRD) patterns of TbBOCl synthesized at 3 days

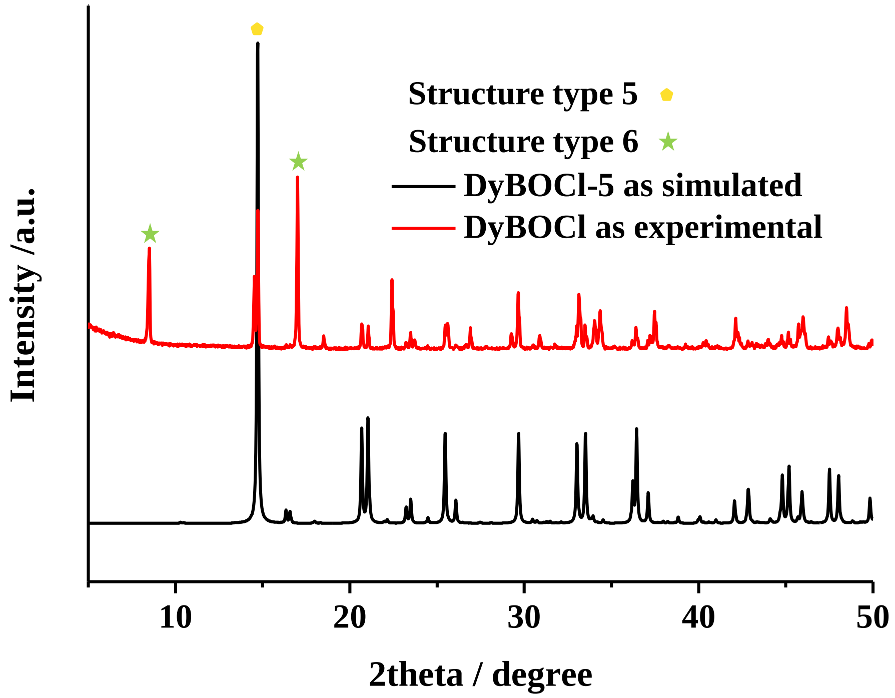

Supplementary Figure 9. Powder X-ray diffraction (PXRD) patterns of DyBOCl synthesized at 3 days

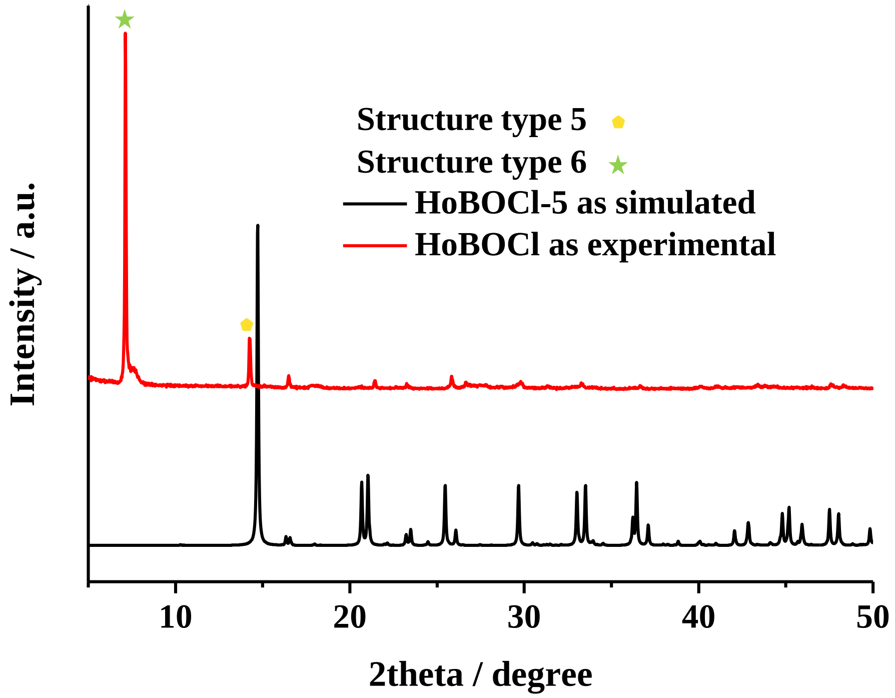

Supplementary Figure 10. Powder X-ray diffraction (PXRD) patterns of HoBOCl synthesized at 3 days

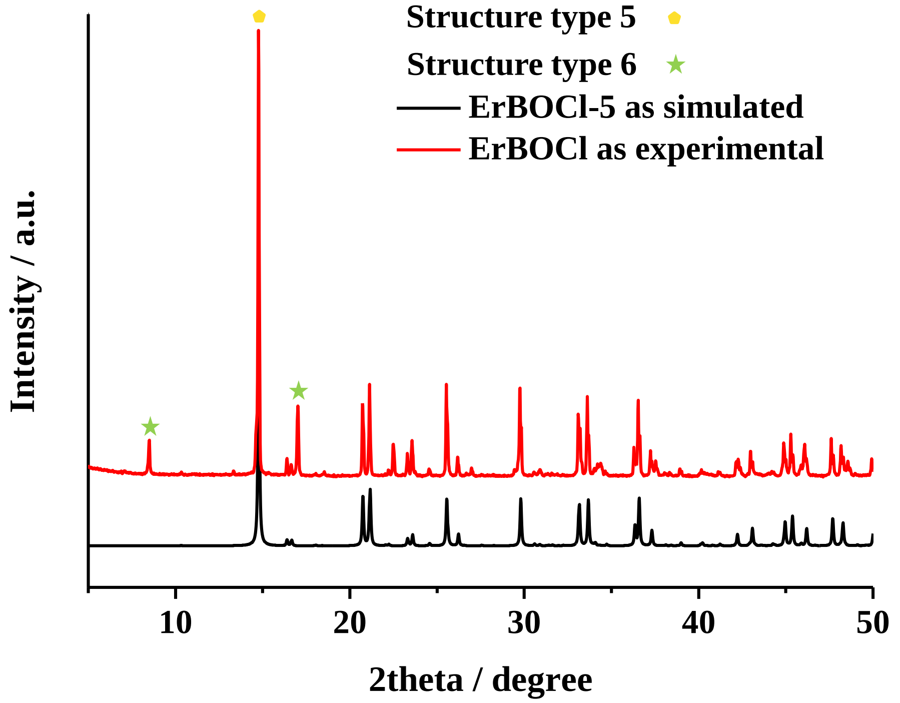

Supplementary Figure 11. Powder X-ray diffraction (PXRD) patterns of ErBOCl synthesized at 3 days

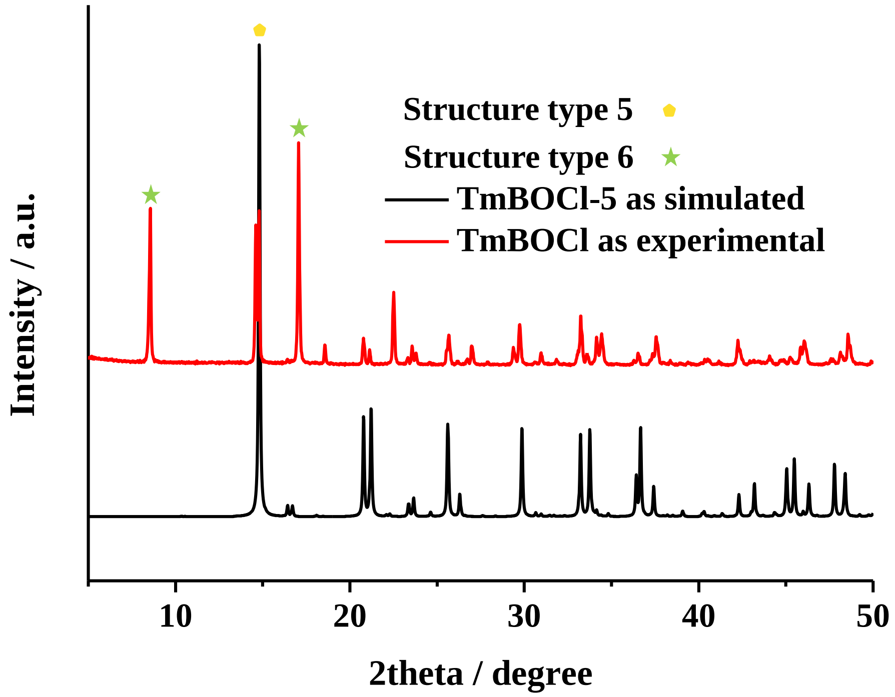

Supplementary Figure 12. Powder X-ray diffraction (PXRD) patterns of TmBOCl synthesized at 3 days

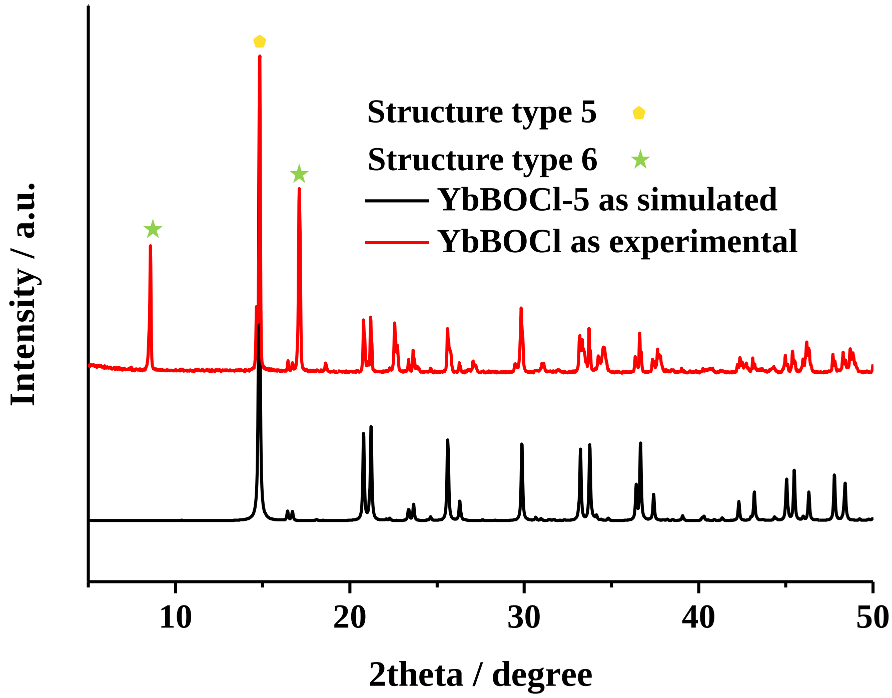

Supplementary Figure 13. Powder X-ray diffraction (PXRD) patterns of YbBOCl synthesized at 3 days

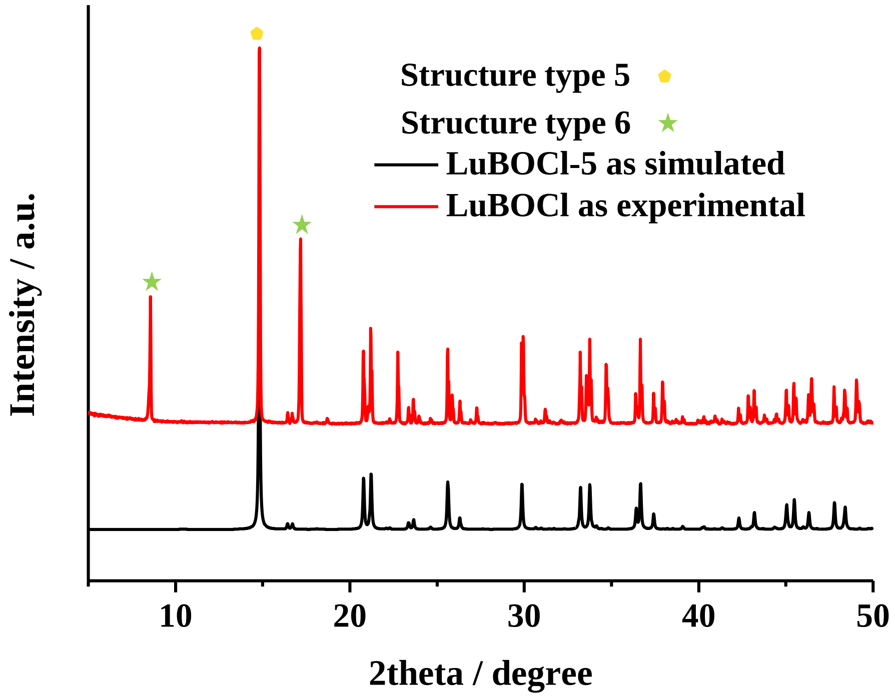

Supplementary Figure 14. Powder X-ray diffraction (PXRD) patterns of LuBOCl synthesized at 3 days

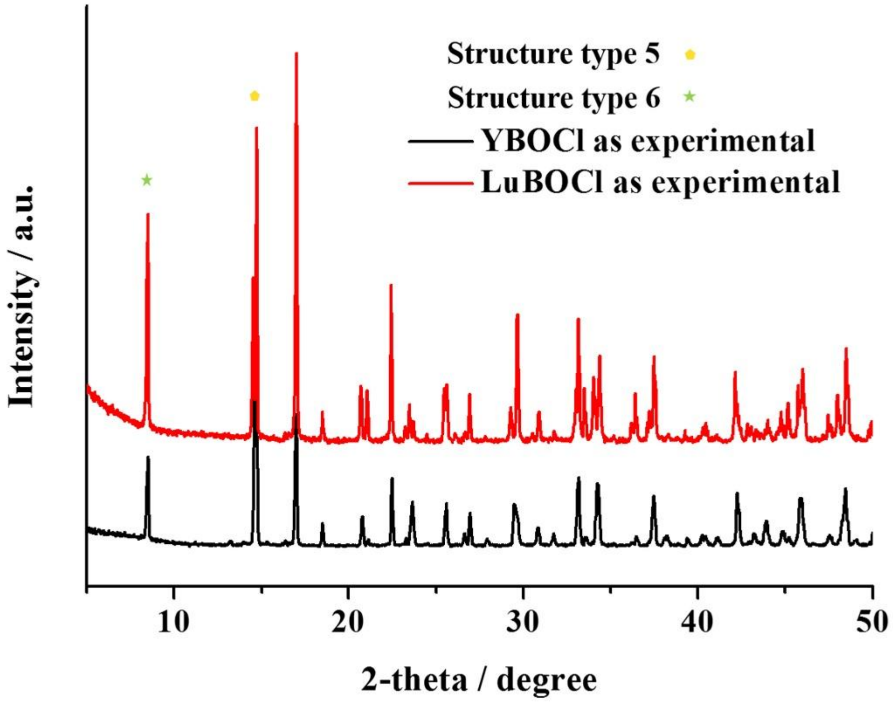

Supplementary Figure 15. Powder X-ray diffraction (PXRD) patterns of YBOCl synthesized at 3 days

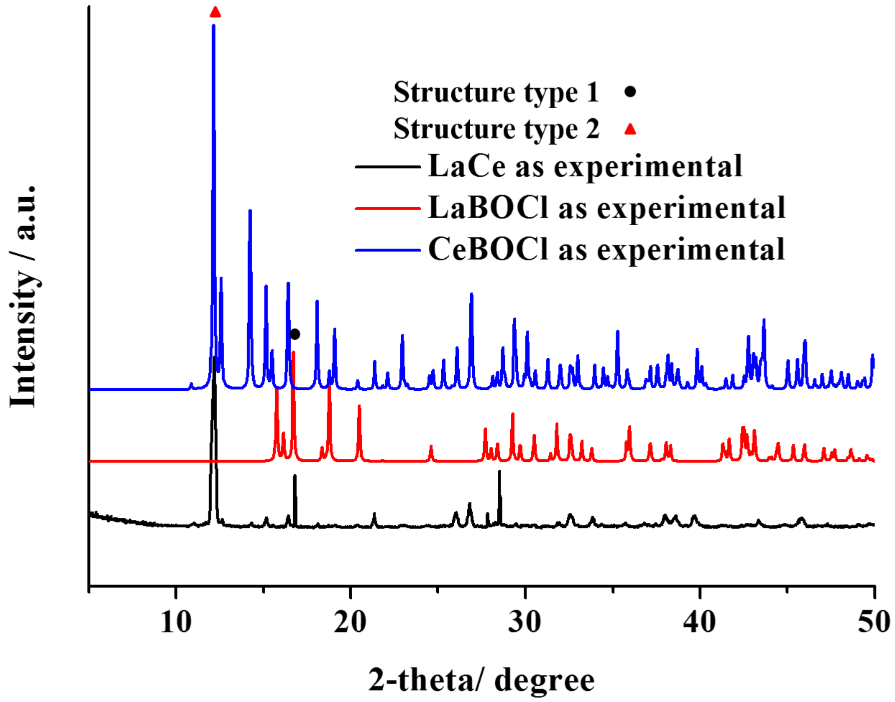

Supplementary Figure 16. Powder X-ray diffraction (PXRD) patterns of La/Ce reaction products synthesized at 3 days

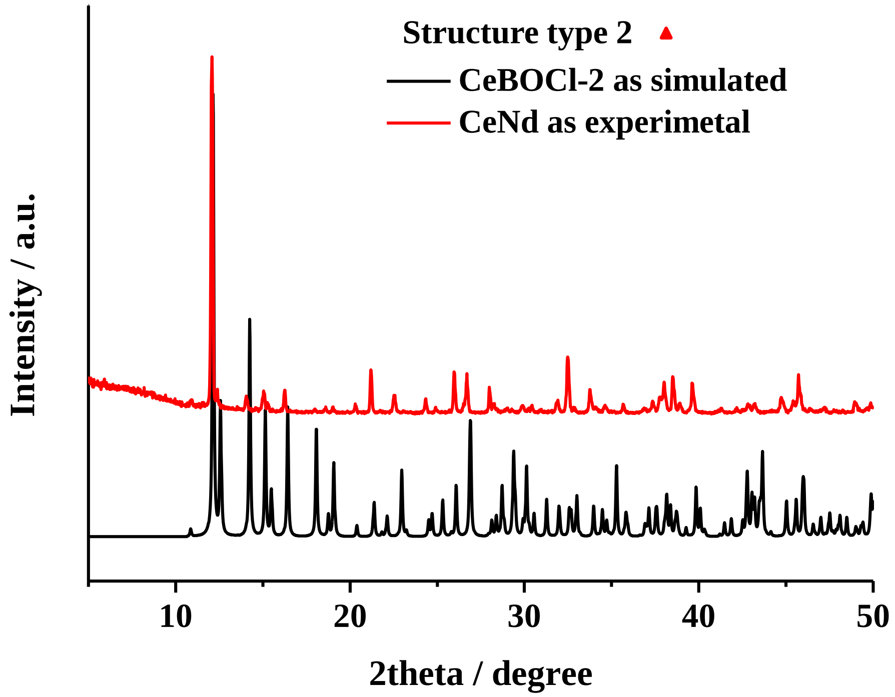

Supplementary Figure 17. Powder X-ray diffraction (PXRD) patterns of Ce/Nd reaction products synthesized at 3 days

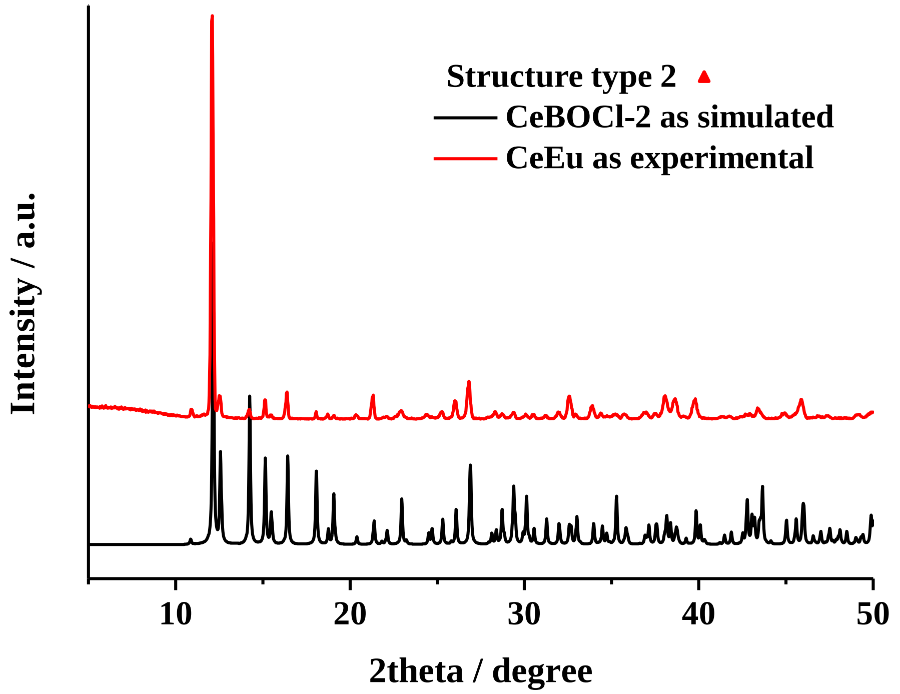

Supplementary Figure 18. Powder X-ray diffraction (PXRD) patterns of Ce/Eu reaction products synthesized at 3 days

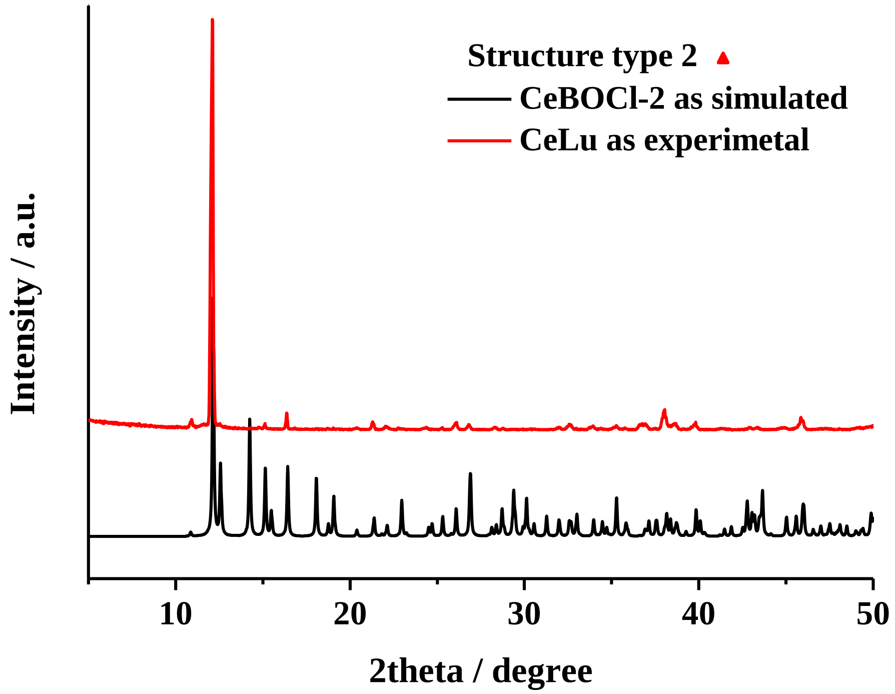

Supplementary Figure 19. Powder X-ray diffraction (PXRD) patterns of Ce/Lu reaction products synthesized at 3 days

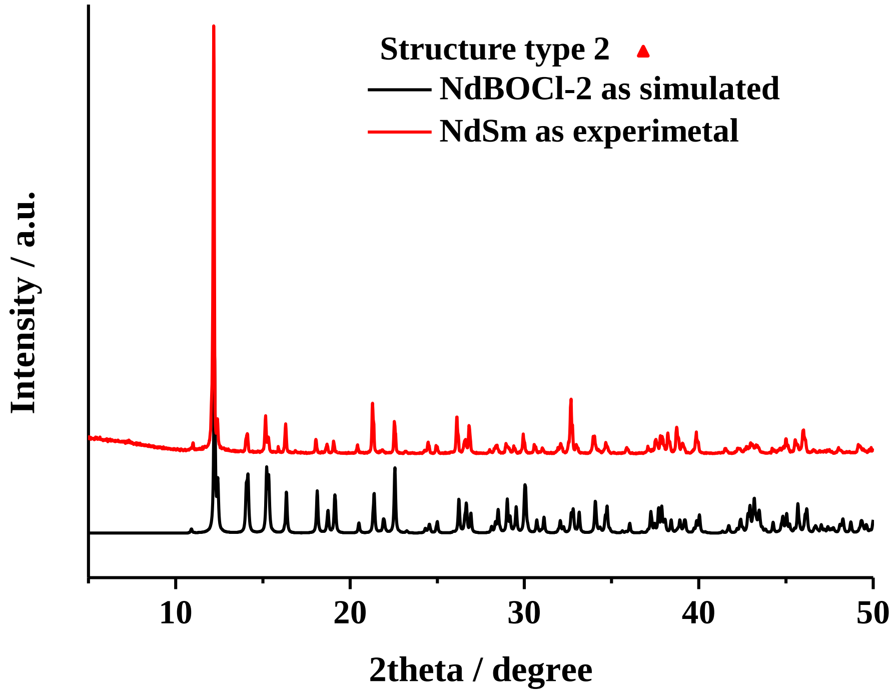

Supplementary Figure 20. Powder X-ray diffraction (PXRD) patterns of Nd/Sm reaction products synthesized at 3 days

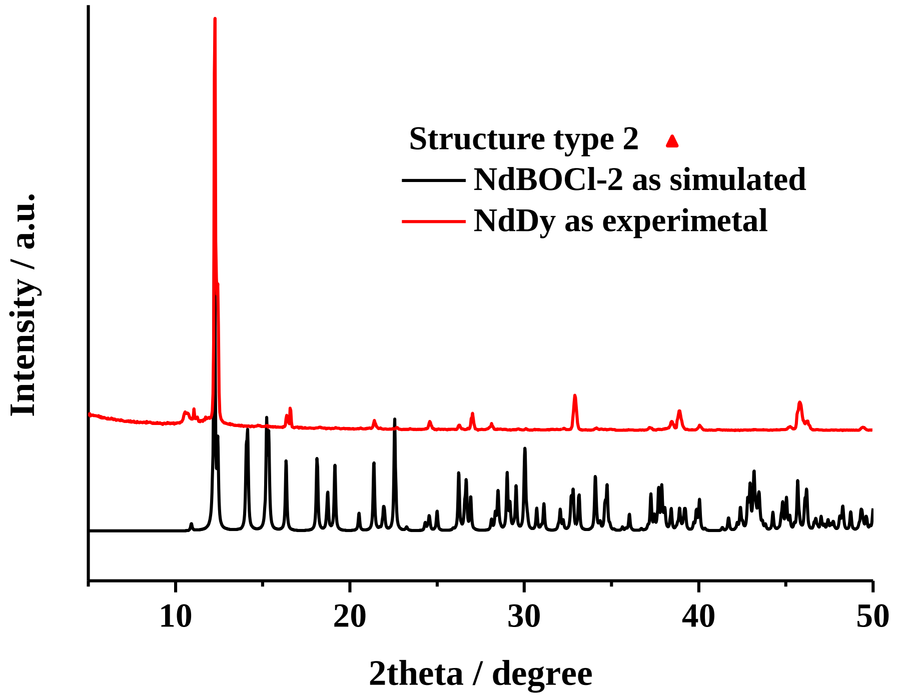

Supplementary Figure 21. Powder X-ray diffraction (PXRD) patterns of Nd/Dy reaction products synthesized at 3 days

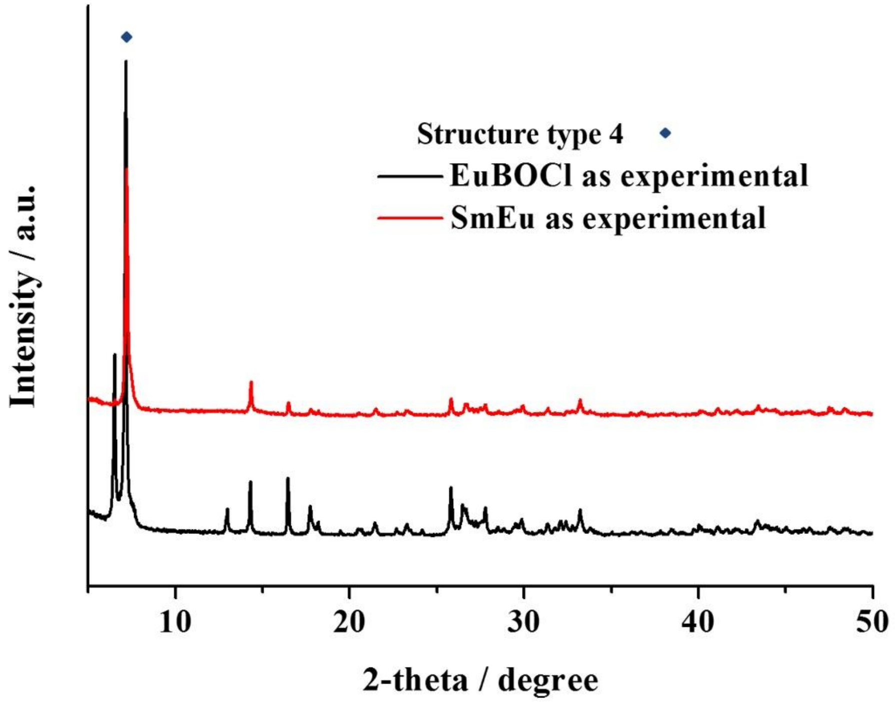

Supplementary Figure 22. Powder X-ray diffraction (PXRD) patterns of Sm/Eu reaction products synthesized at 3 days

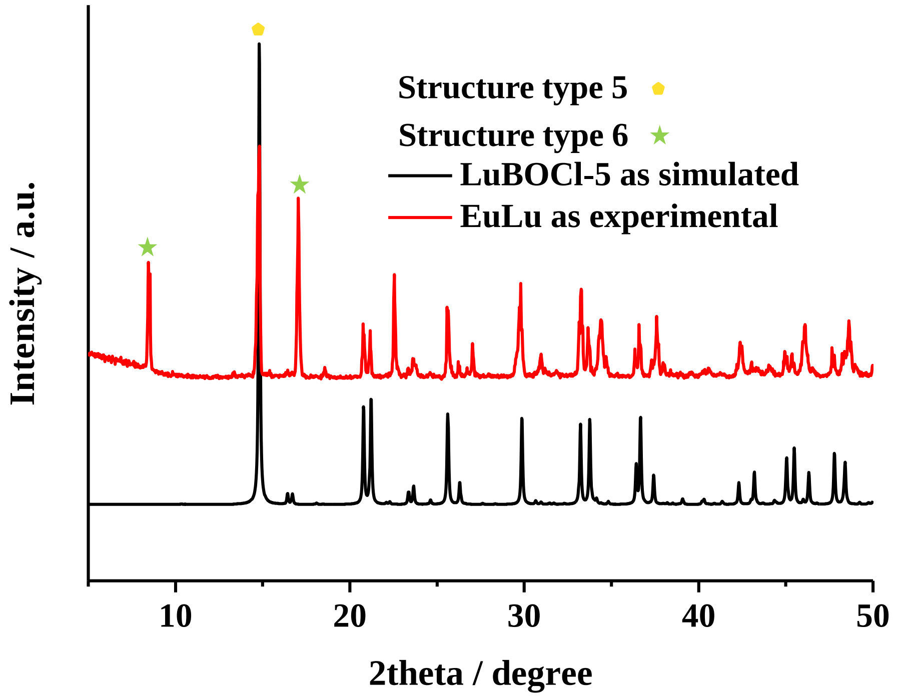

Supplementary Figure 23. Powder X-ray diffraction (PXRD) patterns of Eu/Lu reaction products synthesized at 3 days

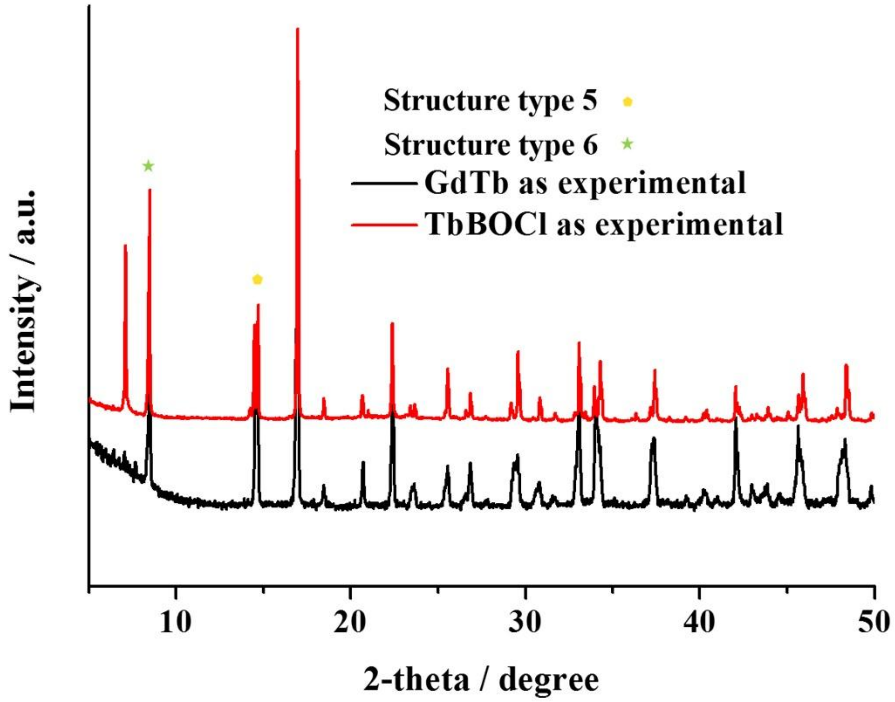

Supplementary Figure 24. Powder X-ray diffraction (PXRD) patterns of Gd/Tb reaction products synthesized at 3 days

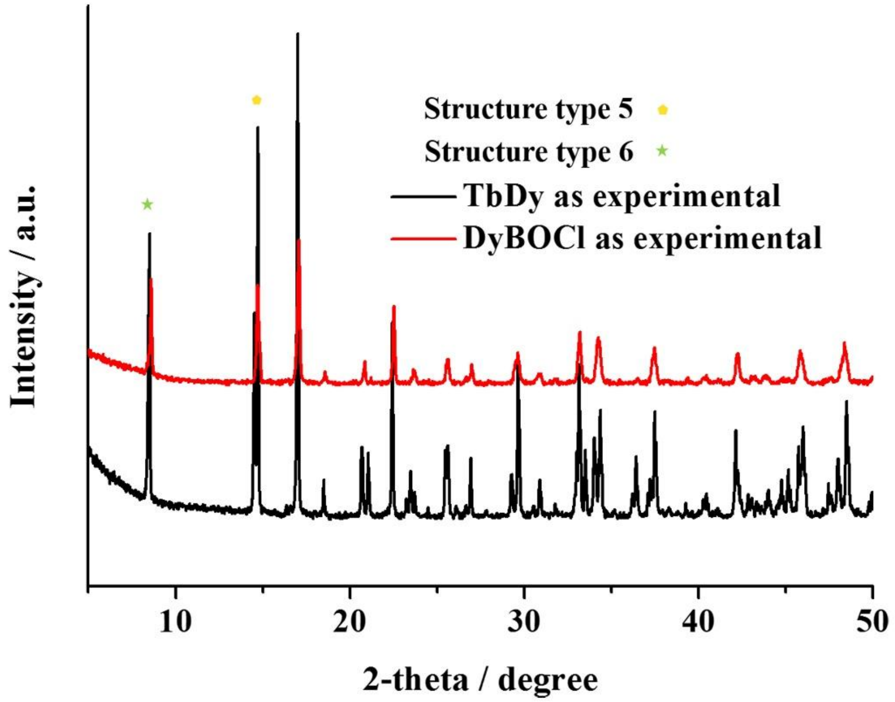

Supplementary Figure 25. Powder X-ray diffraction (PXRD) patterns of Tb/Dy reaction products synthesized at 3 days

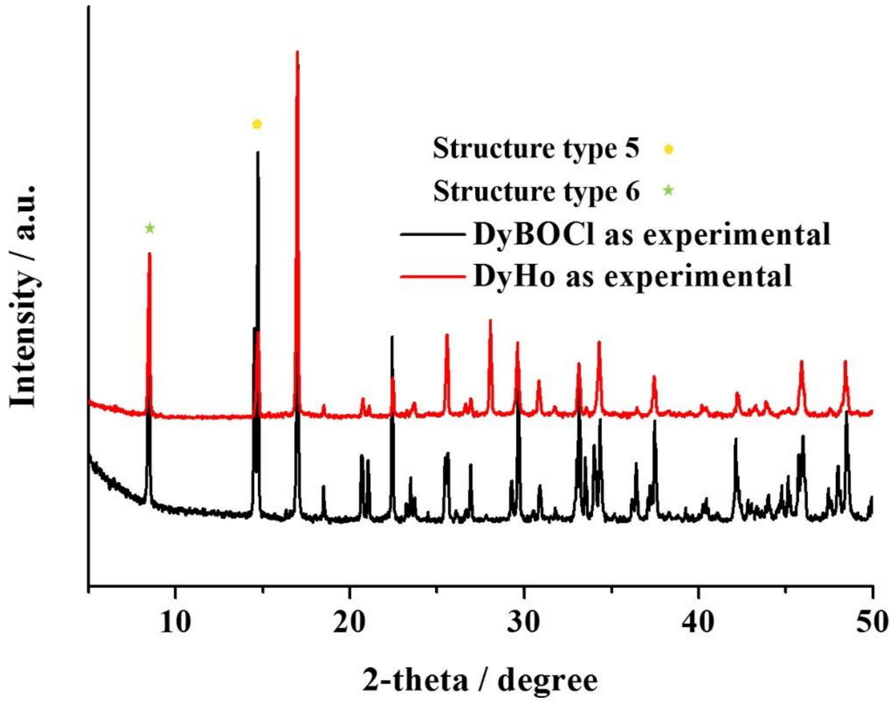

Supplementary Figure 26. Powder X-ray diffraction (PXRD) patterns of Dy/Ho reaction products synthesized at 3 days

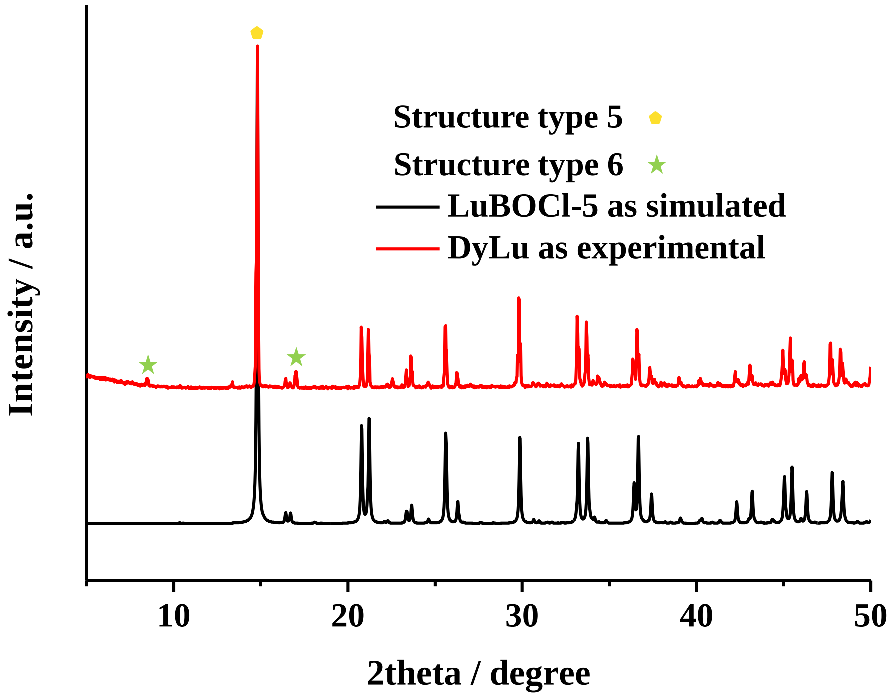

Supplementary Figure 27. Powder X-ray diffraction (PXRD) patterns of Dy/Lu reaction products synthesized at 3 days

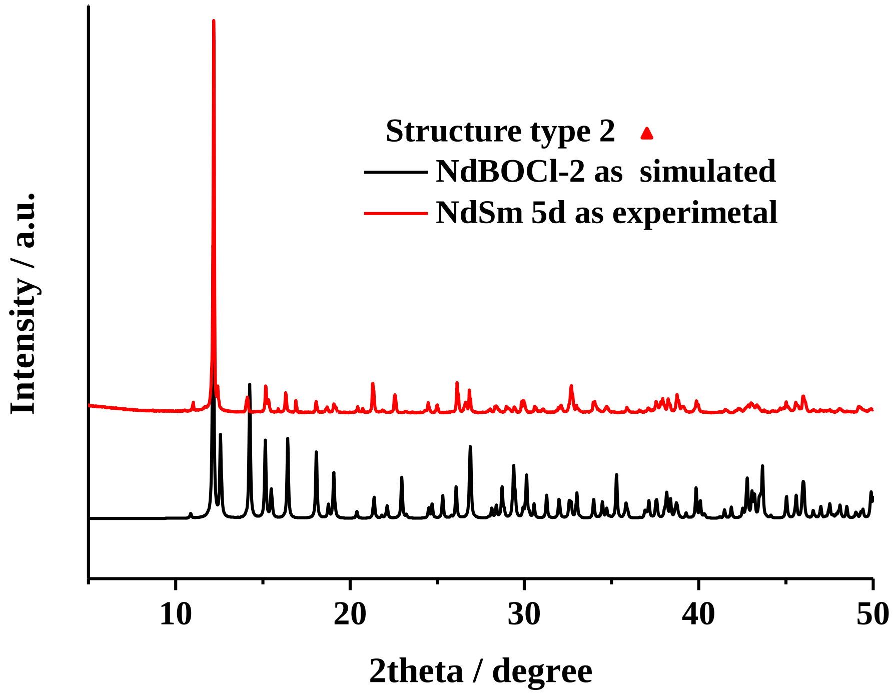

Supplementary Figure 28. Powder X-ray diffraction (PXRD) patterns of Nd/Sm reaction products synthesized at 5 days

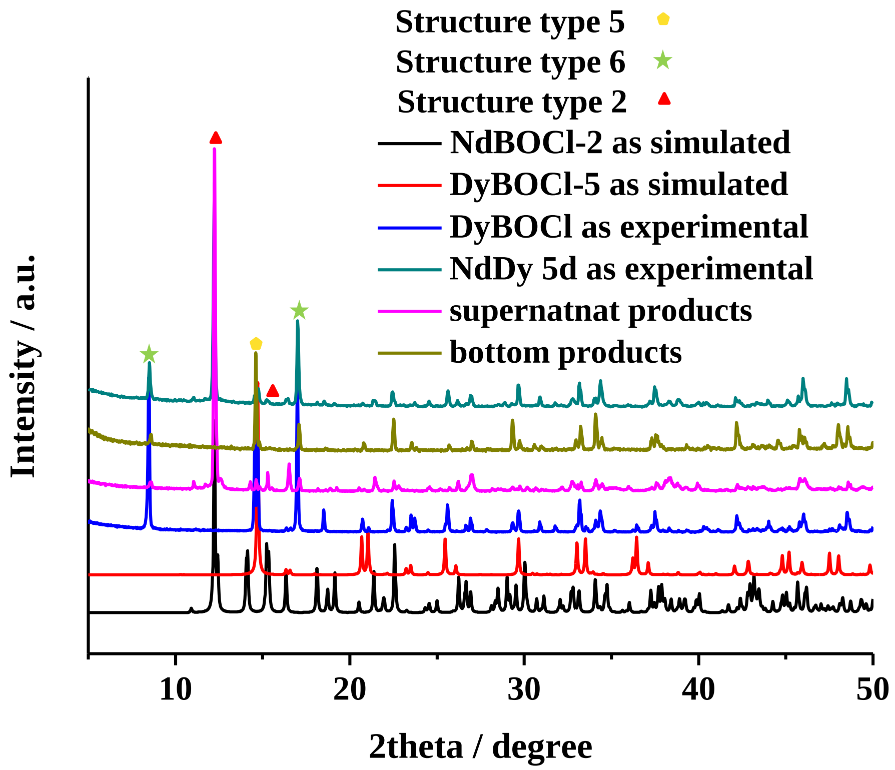

Supplementary Figure 29. Powder X-ray diffraction (PXRD) patterns of Nd/Dy reaction products synthesized at 5 days

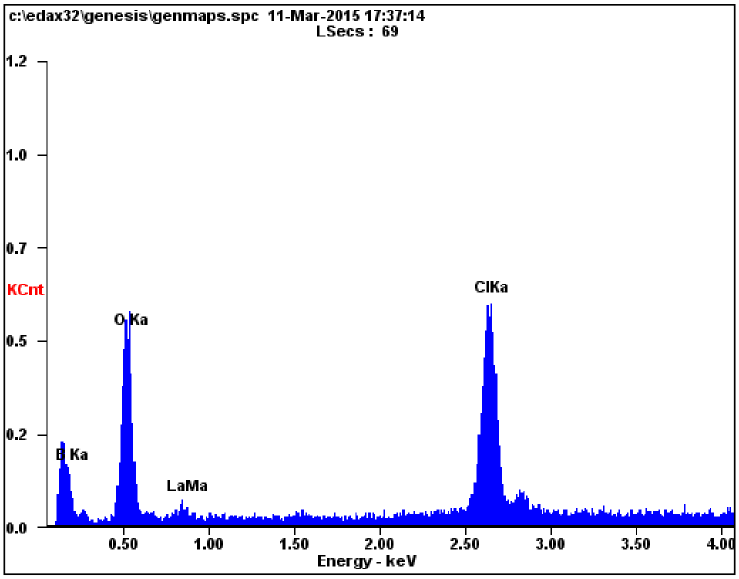

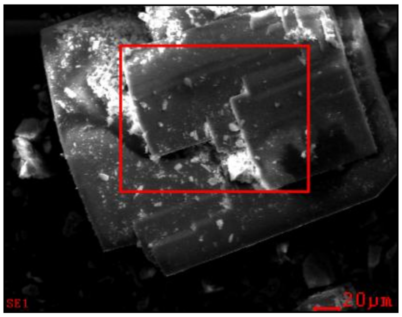

Element	*Wt%*	*At%*
BK	42.78	70.00
OK	21.12	23.35
LaM	30.57	03.89
ClK	05.53	02.76
Matrix	Correction	ZAF

Supplementary Figure 30. The EDS results for LaBOCl-1 crystals synthesized at 3 days

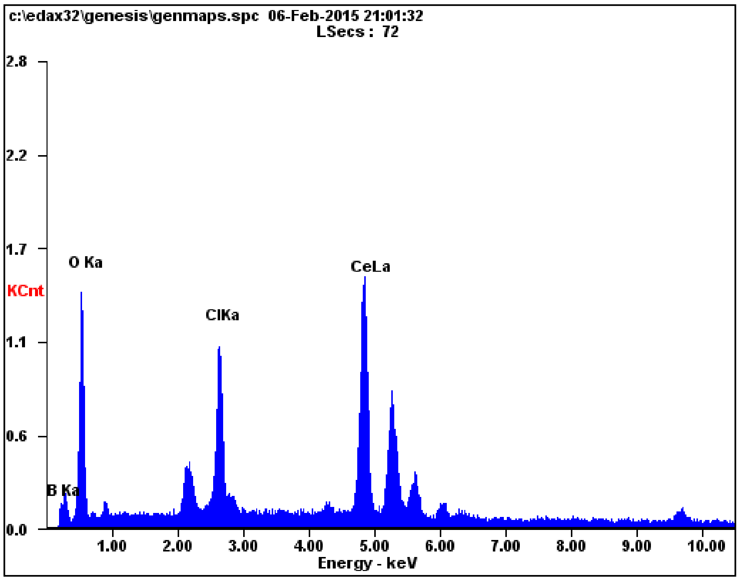

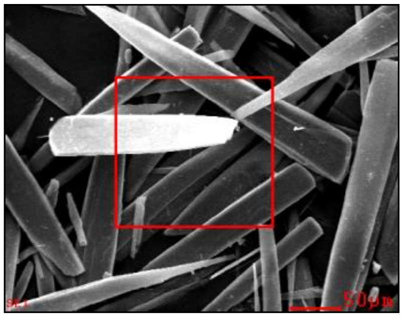

Element	***Wt%***	***At%***
BK	35.16	59.77
OK	29.00	33.31
ClK	05.73	02.97
CeL	30.11	03.95
Matrix	Correction	ZAF

Supplementary Figure 31. The EDS results for CeBOCl-2 crystals synthesized at 3 days

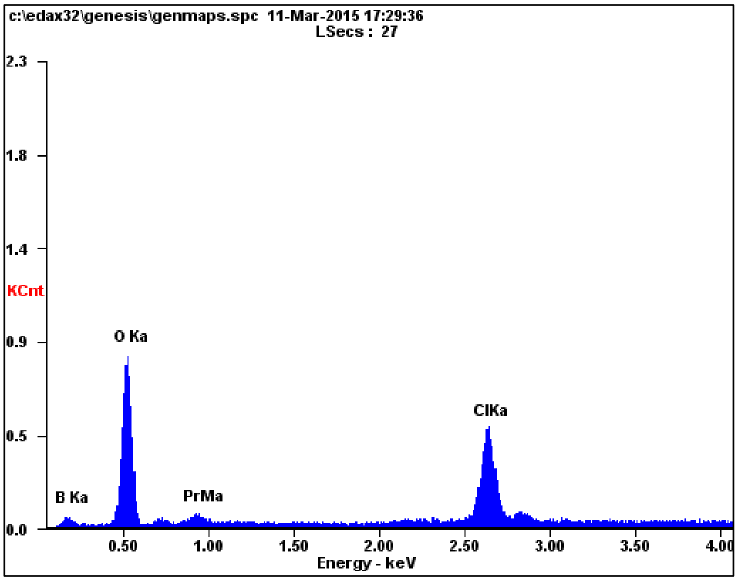

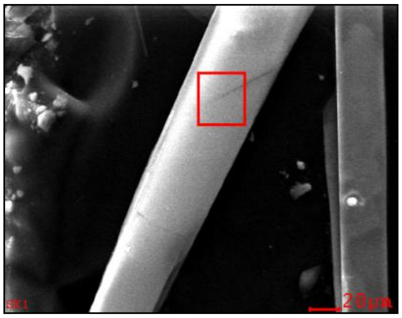

Element	*Wt%*	*At%*
BK	47.36	61.53
OK	40.72	35.75
PrM	06.75	00.67
ClK	05.16	02.05
Matrix	Correction	ZAF

Supplementary Figure 32. The EDS results for PrBOCl-2 crystals synthesized at 3 days

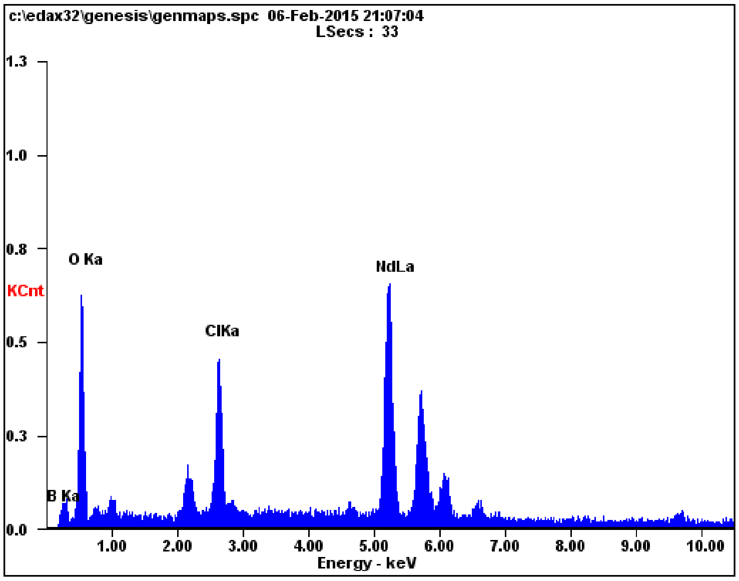

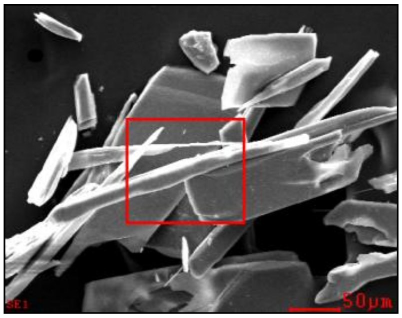

Element	*Wt%*	*At%*
BK	30.37	54.99
OK	30.53	37.36
ClK	05.64	03.12
NdL	33.46	04.54
Matrix	Correction	ZAF

Supplementary Figure 33. The EDS results for NdBOCl-2 crystals synthesized at 3 days

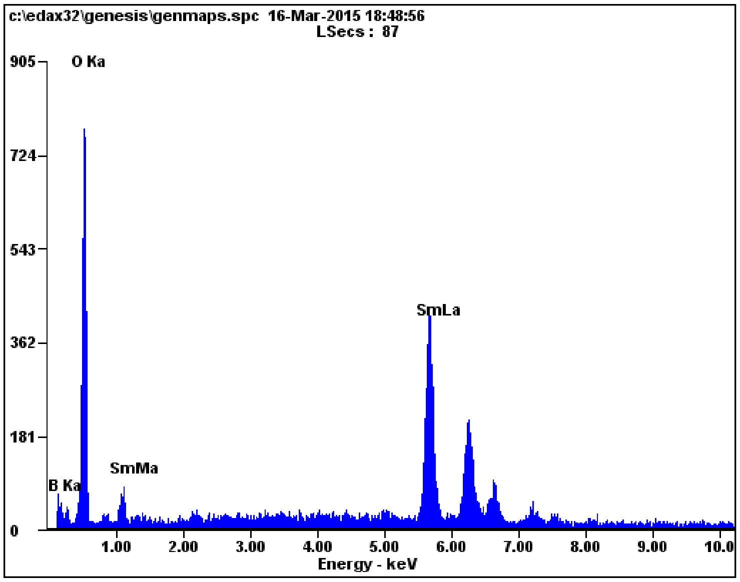

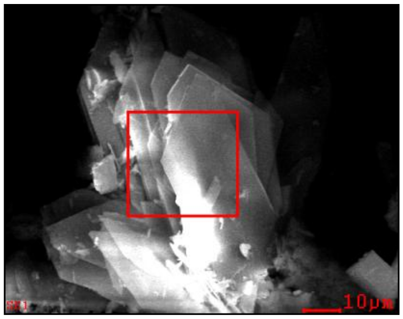

Element	***Wt%***	***At%***
BK	40.21	61.29
OK	34.94	35.98
SmL	24.85	02.72
Matrix	Correction	ZAF

Supplementary Figure 34. The EDS results for SmBOCl-3 crystals synthesized at 3 days

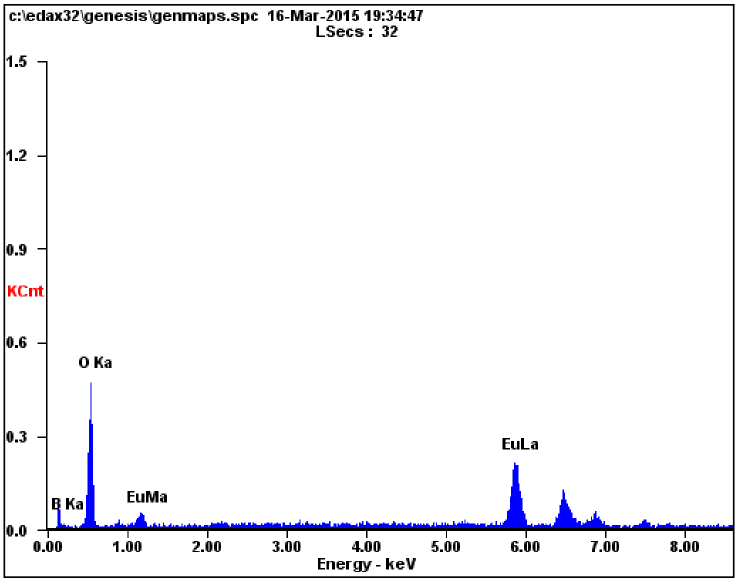

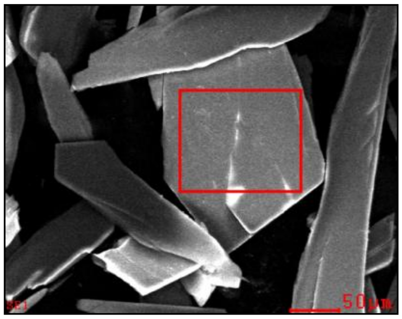

Element	*Wt%*	*At%*
BK	32.46	53.64
OK	38.46	42.95
EuL	29.08	03.42
Matrix	Correction	ZAF

Supplementary Figure 35. The EDS results for EuBOCl-3 crystals synthesized at 3 days

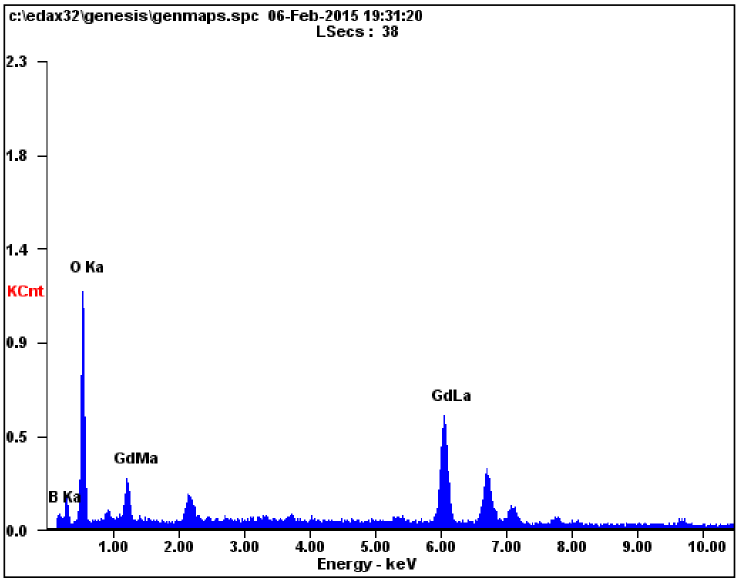

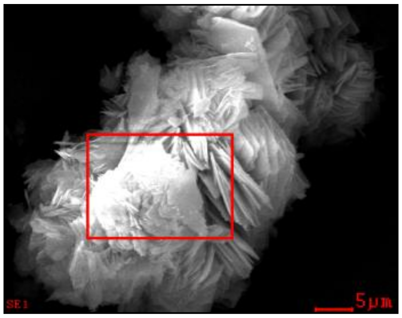

Element	*Wt%*	*At%*
BK	39.08	60.48
OK	35.16	36.78
GdL	25.76	02.74
Matrix	Correction	ZAF

Supplementary Figure 36. The EDS results for GdBOCl-4 crystals synthesized at 3 days

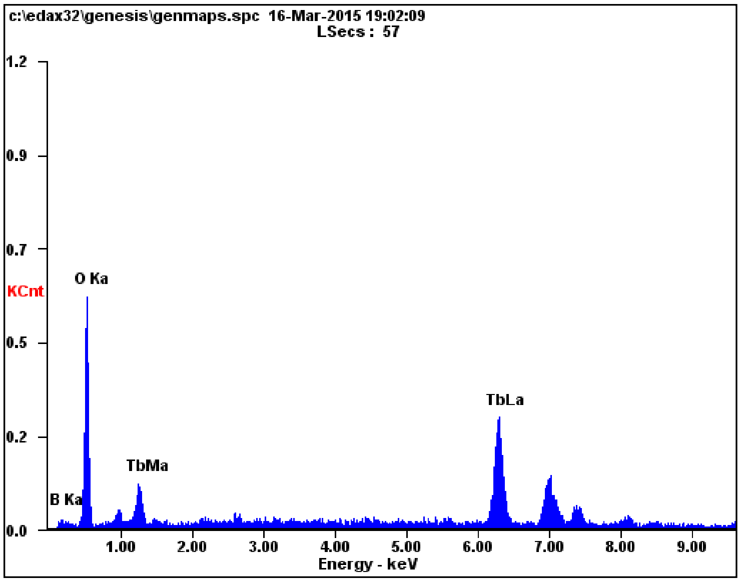

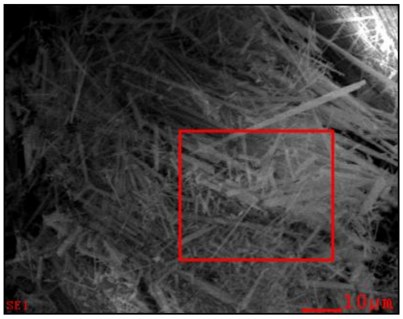

Element	***Wt%***	***At%***
BK	29.91	51.49
OK	38.51	44.81
TbL	31.58	03.70
Matrix	Correction	ZAF

Supplementary Figure 37. The EDS results for TbBOCl-5 crystals synthesized at 3 days

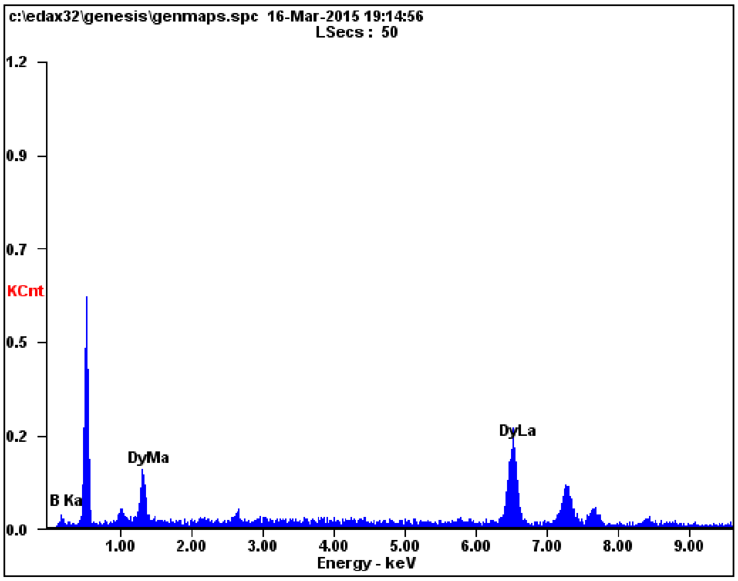

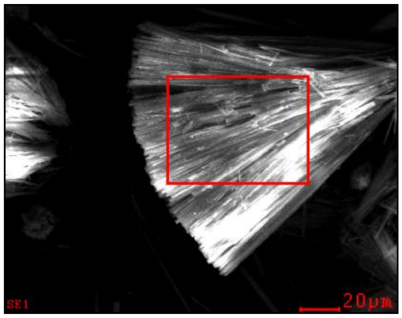

Element	*Wt%*	*At%*
BK	43.59	64.21
OK	33.72	33.57
DyL	22.68	02.22
Matrix	Correction	ZAF

Supplementary Figure 38. The EDS results for DyBOCl-5 crystals synthesized at 3 days

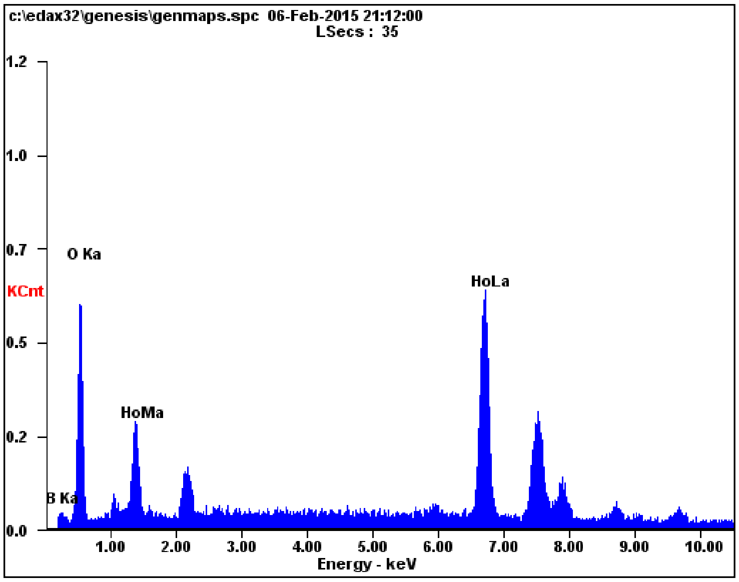

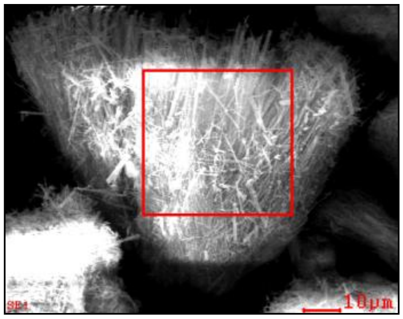

Element	*Wt%*	*At%*
BK	26.66	53.57
OK	29.99	40.72
HoL	43.35	05.71
Matrix	Correction	ZAF

Supplementary Figure 39. The EDS results for HoBOCl-5 crystals synthesized at 3 days

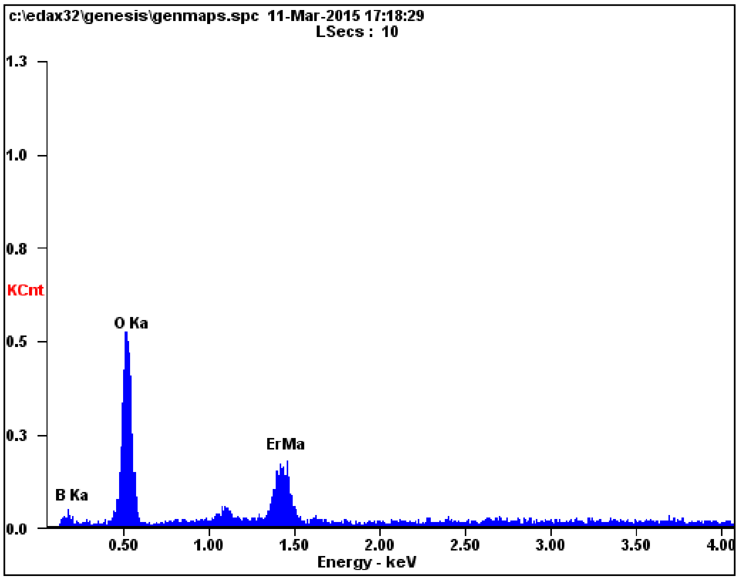

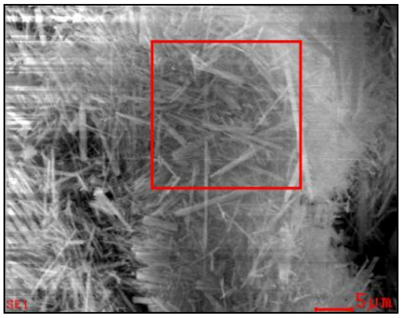

Element	***Wt%***	***At%***
BK	43.45	62.22
OK	37.19	35.99
ErM	19.37	01.79
Matrix	Correction	ZAF

. **Supplementary Figure 40.** The EDS results for ErBOCl-5 crystals synthesized at 3 days

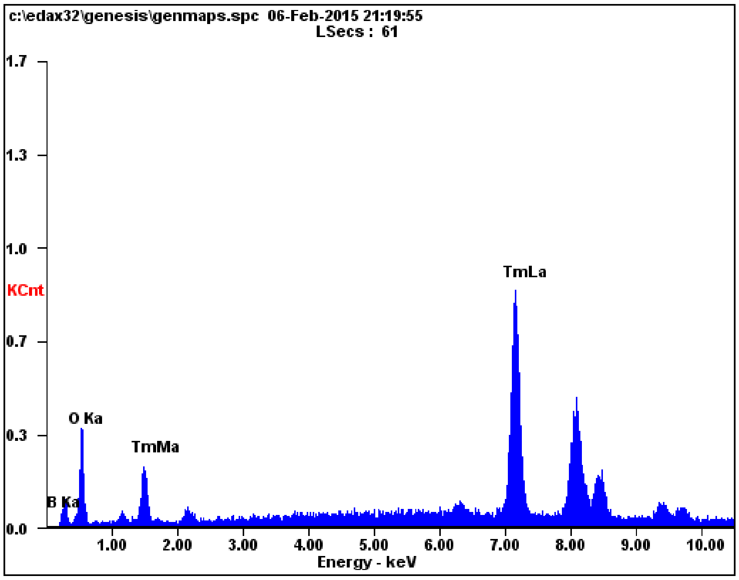

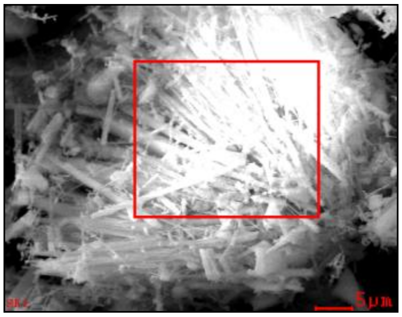

Element	***Wt%***	***At%***
BK	26.56	64.21
OK	16.51	26.98
TmL	56.93	08.81
Matrix	Correction	ZAF

Supplementary Figure 41. The EDS results for TmBOCl-5 crystals synthesized at 3 days

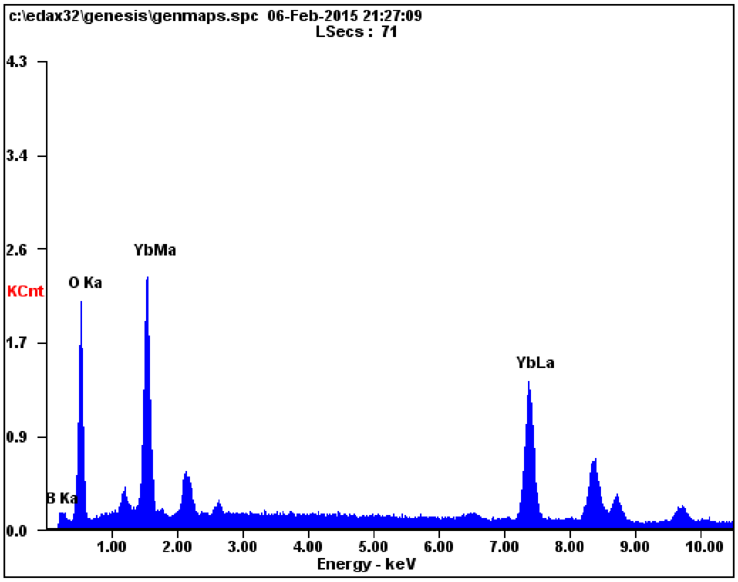

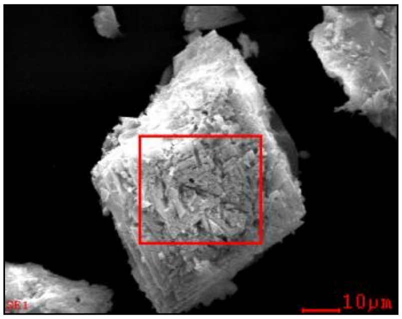

Element	*Wt%*	*At%*
BK	28.83	52.93
OK	34.56	42.87
YbL	36.60	04.20
Matrix	Correction	ZAF

Supplementary Figure 42. The EDS results for YbBOCl-5 crystals synthesized at 3 days

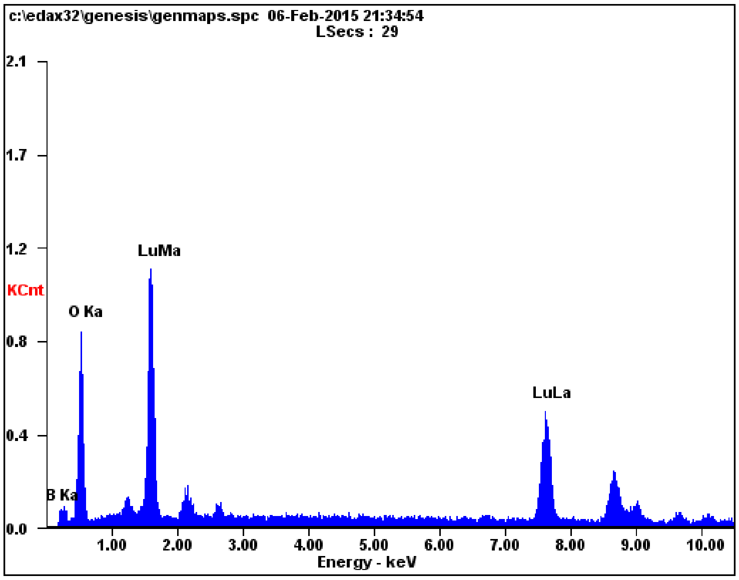

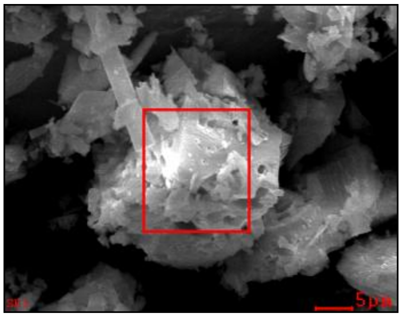

Element	***Wt%***	***At%***
BK	35.27	59.13
OK	33.19	37.60
LuL	31.54	03.27
Matrix	Correction	ZAF

Supplementary Figure 43. The EDS results for LuBOCl-5 crystals synthesized at 3 days

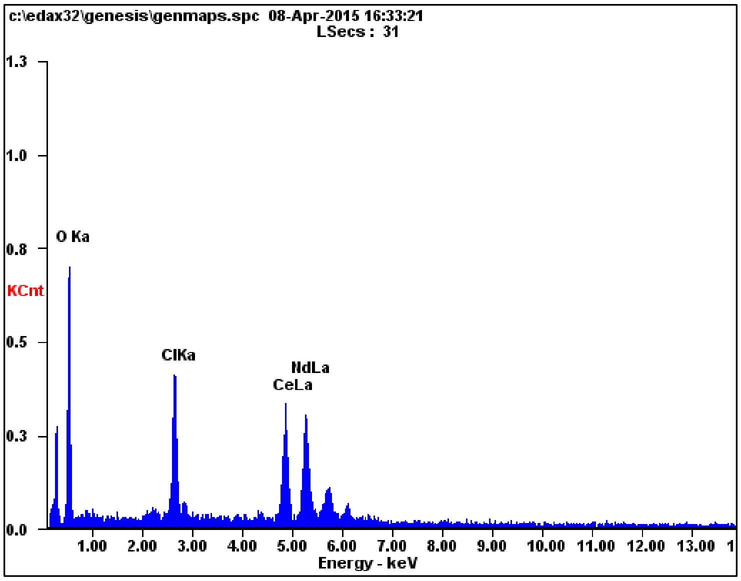

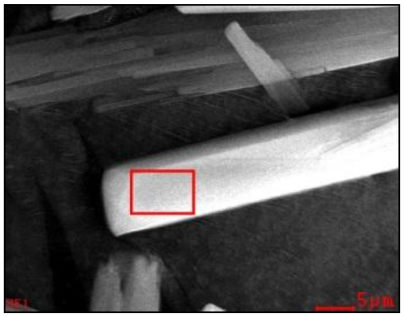

Element	*Wt%*	*At%*
BK	41.49	63.27
OK	30.88	31.82
ClK	04.88	02.27
CeL	14.45	01.70
NdL	08.30	00.95
Matrix	Correction	ZAF

Supplementary Figure 44. The EDS results for Ce/Nd doping crystals synthesized at 3 days

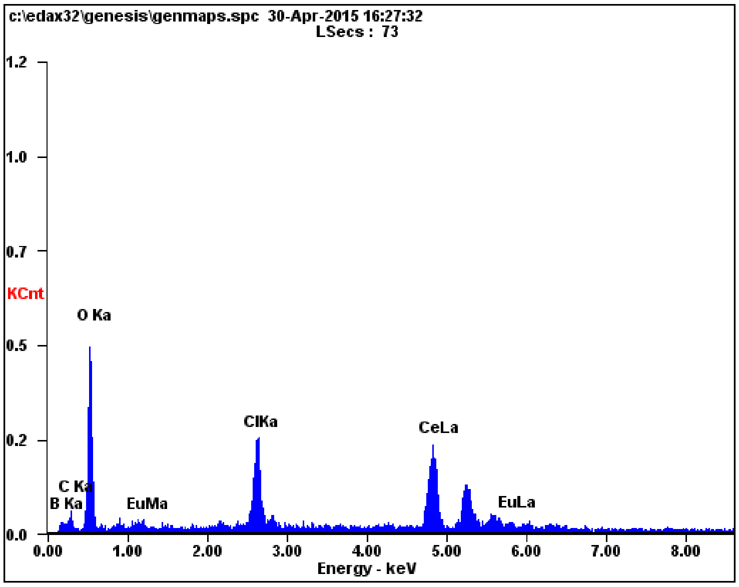

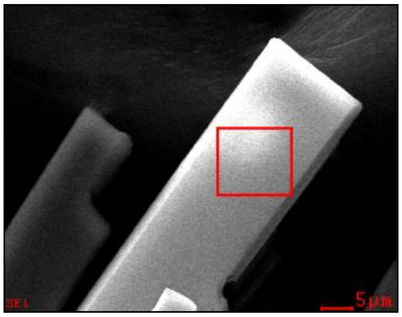

Element	*Wt%*	*At%*
BK	46.95	69.58
OK	25.79	25.83
ClK	04.42	02.00
CeL	21.30	02.44
EuL	01.54	00.16
Matrix	Correction	ZAF

Supplementary Figure 45. The EDS results for Ce/Eu doping crystals synthesized at 3 days

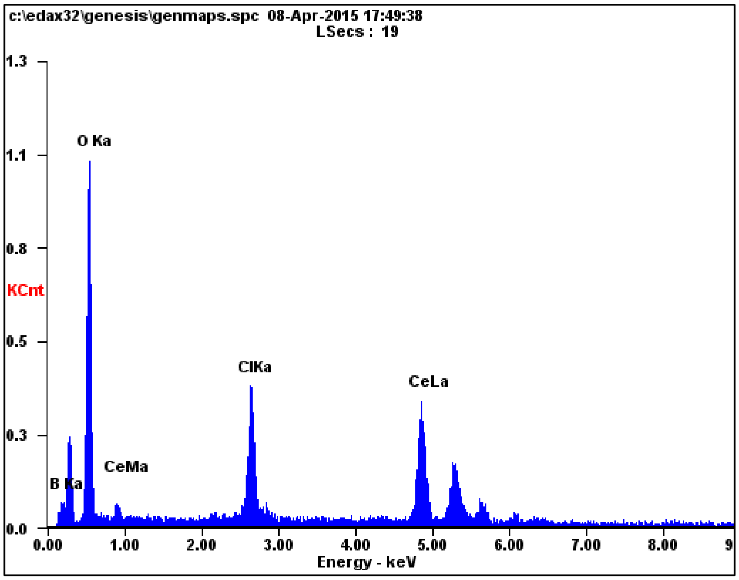

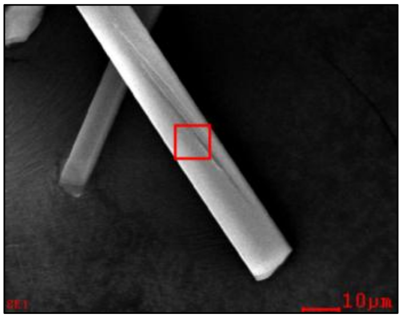

Element	*Wt%*	*At%*
BK	47.78	64.82
OK	35.38	32.44
ClK	03.17	01.31
CeL	13.67	01.43
Matrix	Correction	ZAF

. **Supplementary Figure 46.** The EDS results for Ce/Lu doping crystals synthesized at 3 days

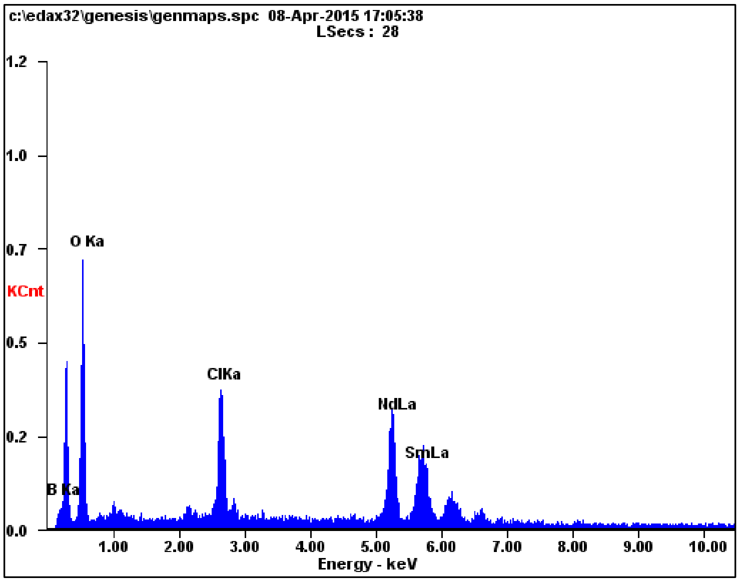

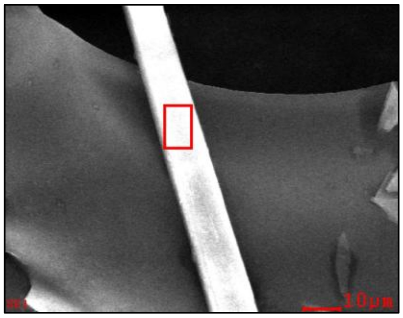

Element	Wt%	At%
BK	43.27	64.78
OK	30.47	30.82
ClK	04.32	01.97
NdL	15.80	01.77
SmL	06.14	00.66
Matrix	Correction	ZAF

Supplementary Figure 47. The EDS results for Nd/Sm doping crystals synthesized at 3 days

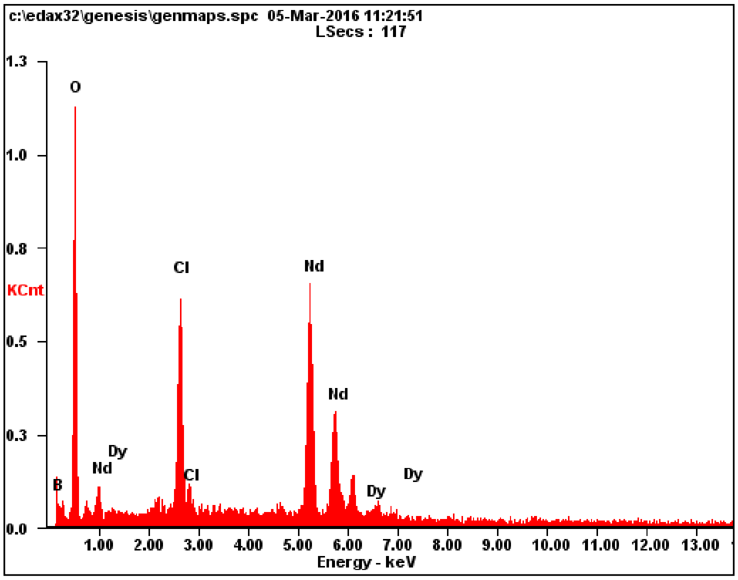

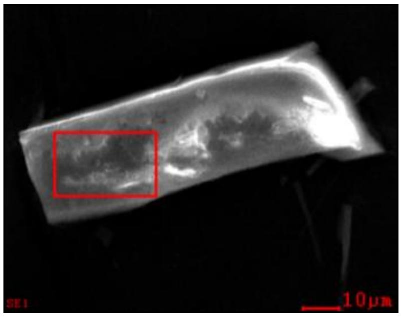

Element	Wt%	At%
BK	45.09	64.76
OK	32.51	31.55
ClK	03.88	01.70
NdL	17.79	01.92
DyL	00.73	00.07
Matrix	Correction	ZAF

Supplementary Figure 48. The EDS results for Nd/Dy doping crystals synthesized at 3 days

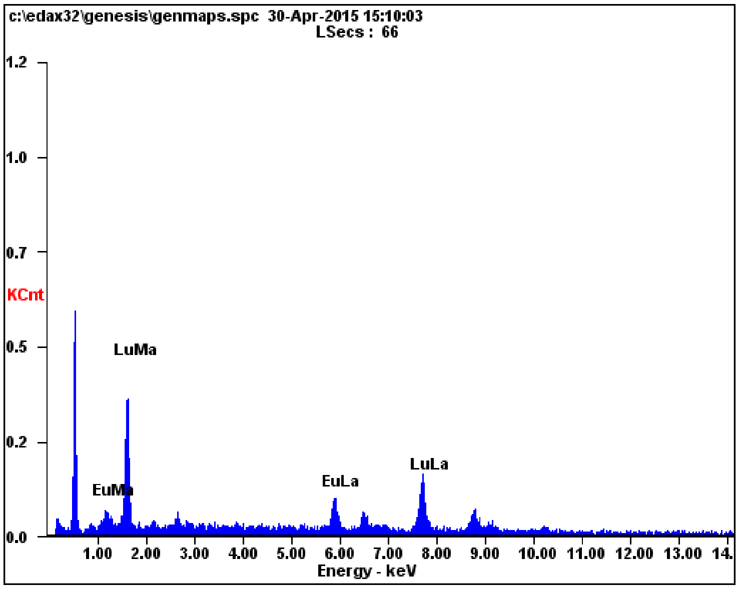

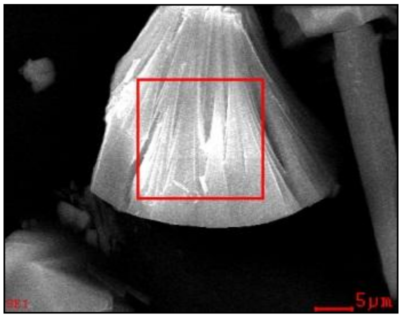

Element	*Wt%*	*At%*
BK	44.74	65.65
OK	32.48	32.20
EuL	05.88	00.61
LuL	16.89	01.53
Matrix	Correction	ZAF

. **Supplementary Figure 49.** The EDS results for Eu/Lu doping crystals synthesized at 3 days

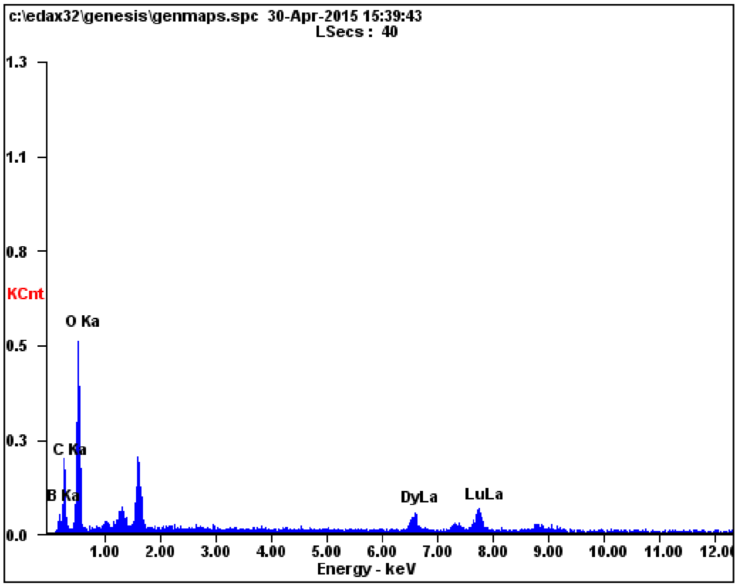

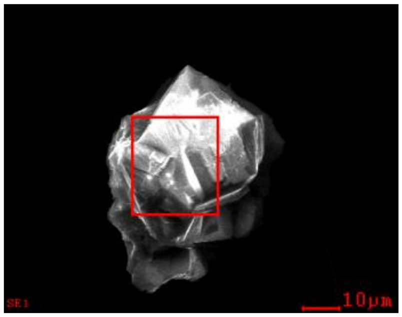

Element	*Wt%*	*At%*
BK	48.15	68.81
OK	30.27	29.23
DyL	08.49	00.81
LuL	13.09	01.16
Matrix	Correction	ZAF

. **Supplementary Figure 50.** The EDS results for Dy/Lu doping crystals synthesized at 3 days

a

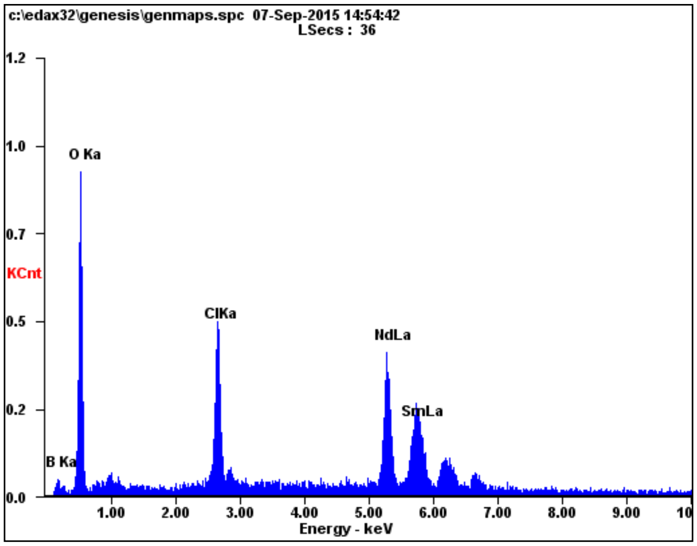

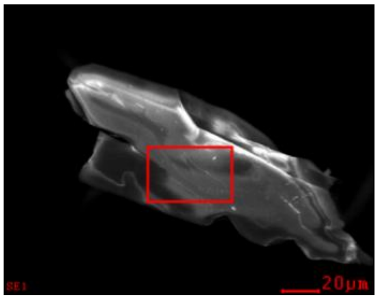

Element	Wt%	At%
BK	36.16	58.13
OK	33.37	36.25
ClK	05.36	02.63
NdL	18.20	02.19
SmL	06.91	00.80
Matrix	Correction	ZAF

b

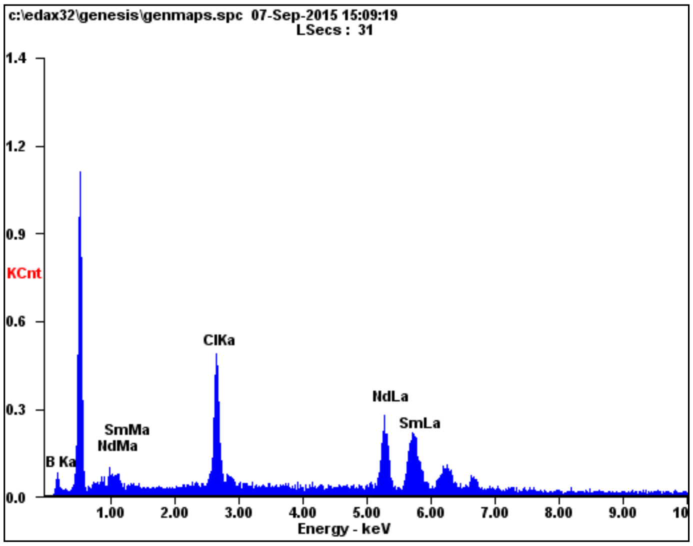

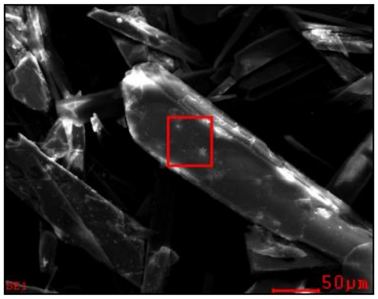

Element	Wt%	At%
BK	43.75	62.04
OK	36.05	34.55
ClK	03.96	01.71
NdL	09.43	01.00
SmL	06.81	00.69
Matrix	Correction	ZAF

Supplementary Figure 51. The EDS results for Nd/Sm doping crystals synthesized at 5 days

a

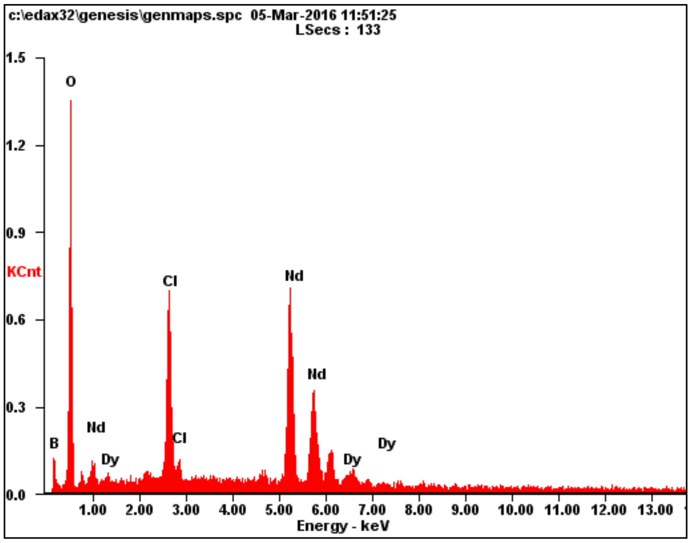

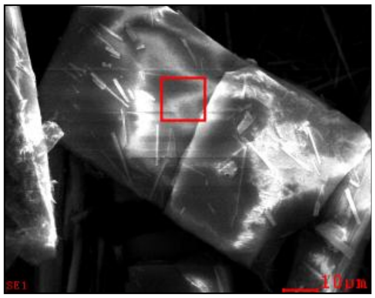

Element	*Wt%*	*At%*
BK	51.22	69.65
OK	29.88	27.45
ClK	03.14	01.30
NdL	14.91	01.52
DyL	00.85	00.08
Matrix	Correction	ZAF

b

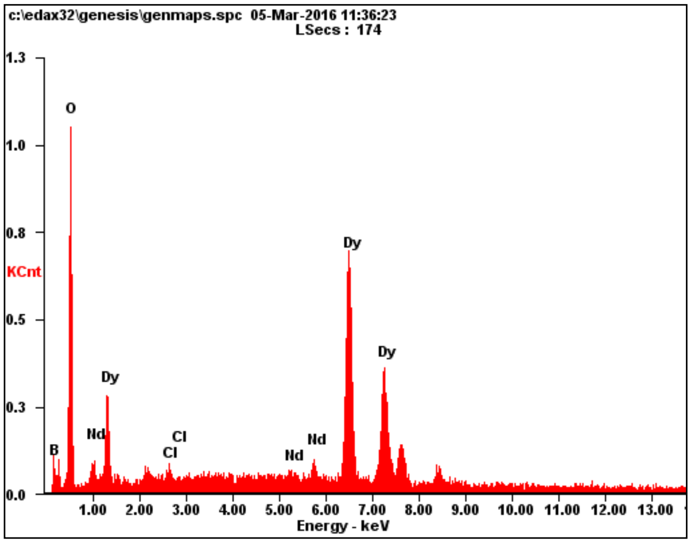

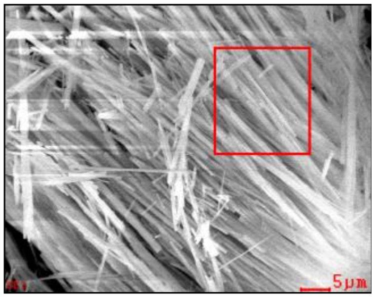

Element	*Wt%*	*At%*
BK	43.38	66.37
OK	29.80	30.81
ClK	00.24	00.11
NdL	00.70	00.08
DyL	25.87	02.63
Matrix	Correction	ZAF

Supplementary Figure 52. The EDS results for Nd/Dy doping crystals synthesized at 5 days

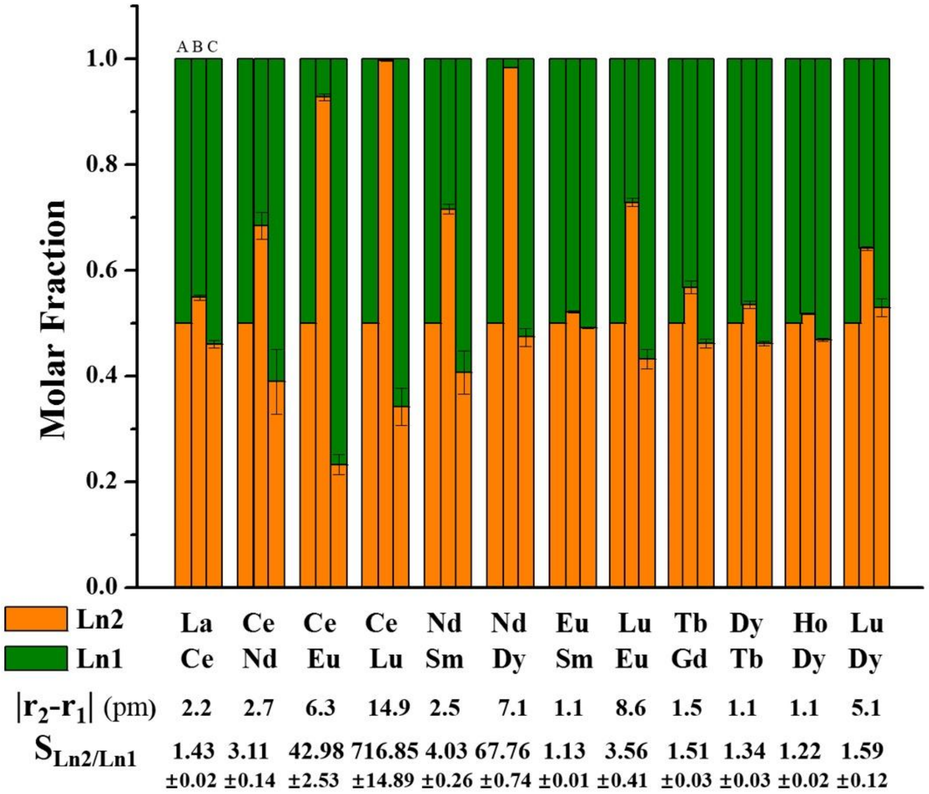

Supplementary Figure 53. Separation results for 12 combinations of Ln1 and Ln2 measured by ICP-MS. (A, B, and C columns represent the molar distribution of Ln1/Ln2 in the starting material, solid products, and wash solutions, receptively). r_2-r_1 represents the difference of Ln^{3+} ionic radius (6-coordinate). $S_{Ln2/Ln1}$ is the seapration factor.

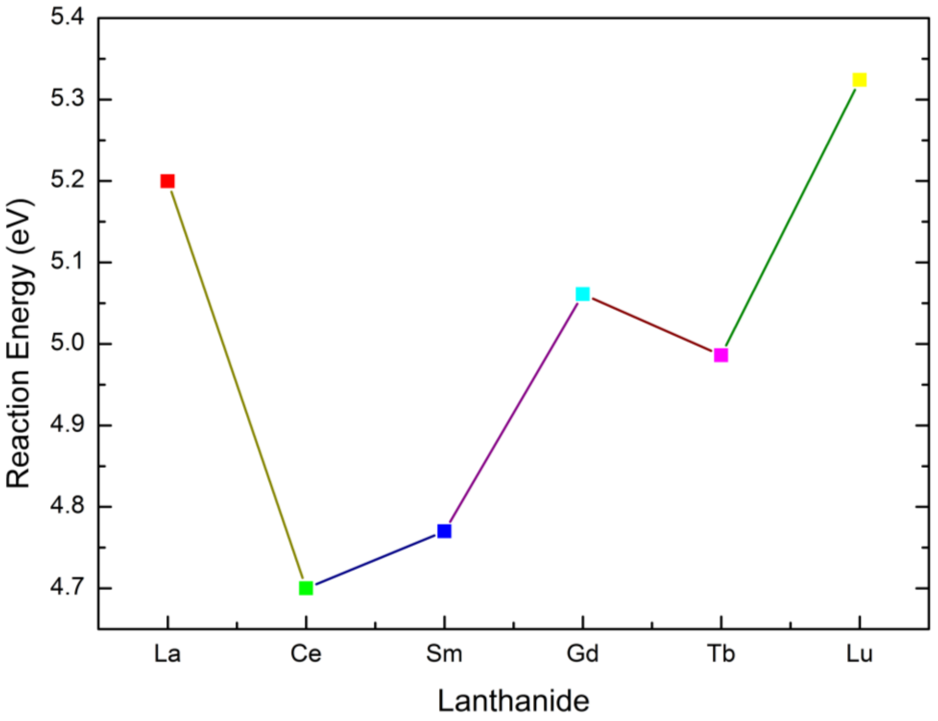

(a)

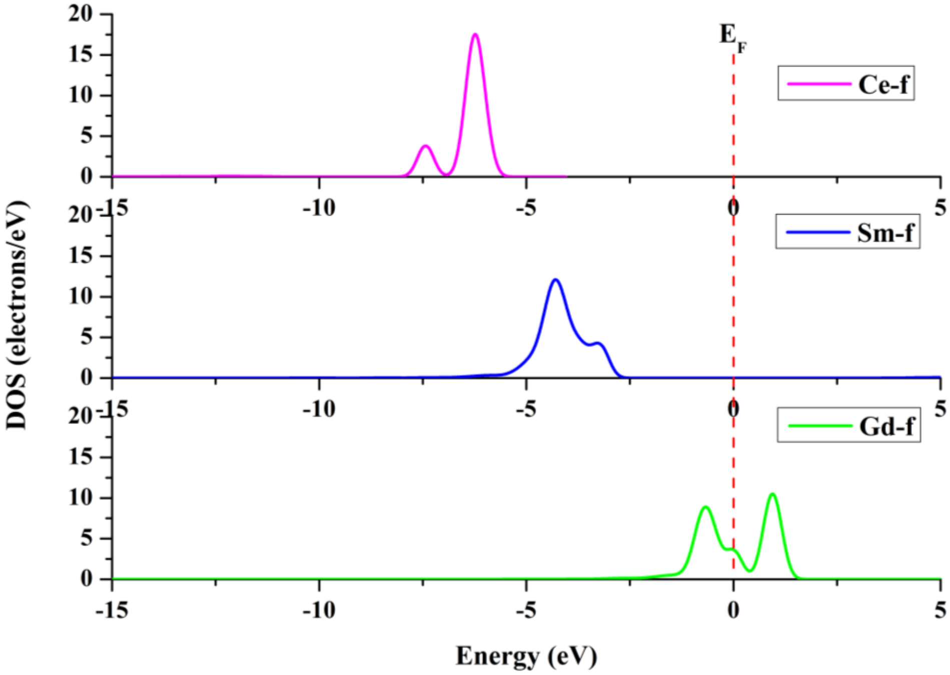

(b)

Supplementary Figure 54. (a) the reaction energy to form structure type 2 for different lanthanides (La, Ce, Sm, Gd, Td, and Lu); (b) The PDOS for 4*f* orbitals in different lanthanides (Ce, Sm, and Gd) borates in structure type 2. By the first principles calculations, the reaction energies for LnBOCl-2 with Ln = La, Ce, Sm, Gd, Tb, Lu as the

examples are predicted in this work as shown in (a). The borate of Ce is determined to be the most stable among the selected ones with 0.49 eV higher in reaction energy than its neighbor La. The difference is 0.10 eV relative to Sm, and increases to over 0.60 eV to Lu. These are the reflection that the difference in lattice energies of lanthanide borates is amplified since the difference in lanthanide atomic radius is subtle (i.e. the well-known lanthanide contraction). The Density of States (DOS) of LnBOCl-2 with Ln = Ce, Sm, and Gd are calculated as well with data shown in (b). Firstly, the electronic structure of LaBOCl-2 is apparently different from others due to the lack of *f* electron. The DOS for 4*f* electrons of the borates of Ce, Sm and Gd exhibit *f* orbital peaks in different shapes below or crossing the Fermi level, indicating the degenerate inner orbitals are splitted by the ligands. It can be found the *f* orbitals for CeBOCl-2 show a high orbital energy of 5.1-7.6 eV below Fermi level, meaning the corresponding product is probably the least influenced. The *f* orbital energy for the borate of Sm appears in the range of 2.8-6.0 eV from the Fermi level. As to Gd, the orbital energy crosses the Fermi level, showing highest reactivity. Thus, one can see that the inner orbital is unevenly influenced and thus the ligand-center atomic interaction show different behaviors since the *s* and *d* electrons of the center atoms are ionized. Consequently, the LnBOCl-2 compounds of different lanthanide elements may exhibit diverse chemical features including activity and formation energies, which may result in difference in apparent separation behavior with borates

Supplementary Table

Supplementary Table 1. Crystallographic data for $LaB_4O_6(OH)_2Cl$ (structure type 1), $Nd_2B_{12}O_{18}Cl_2(OH)_4(H_2O)_4 \cdot H_2O$ (structure type 2), $SmB_6O_8(OH)_5 \cdot H_3BO_3 \cdot H_2O$ (structure type 3), $EuB_6O_8(OH)_5 \cdot H_3BO_3$ (structure type 4), $Lu_4B_{24}O_{36}(OH)_{12} \cdot H_2O$ (structure type 5), and LuBOCl-6 (structure type 6)

compound	$LaB_4O_6(OH)_2Cl$ (structure type 1)	$Nd_2B_{12}O_{18}Cl_2(OH)_4(H_2O)_4 \cdot H_2O$ (structure type 2)	$SmB_6O_8(OH)_5 \cdot H_3BO_3 \cdot H_2O$ (structure type 3)	$EuB_6O_8(OH)_5 \cdot H_3BO_3$ (structure type 4)	$Lu_4B_{24}O_{36}(OH)_{12} \cdot H_2O$ (structure type 5)	LuBOCl-6 (structure type 6)*
Mass	345.60	953.10	498.02	244.46	1743.32	201.78
Color and habit	Colorless. block	Light purple, Needle	Colorless, Tablet	Colorless, plate	Colorless, block	Colorless, Needle
Spaces group	Cc	P2(1)/n	C2/m	P-1	R-3	P3
a(Å)	6.5268(17)	8.1867(4)	8.7754(17)	6.8148(8)	17.0783(4)	11.9186(3)
b(Å)	11.251(3)	14.5421(7)	27.454(5)	7.1444(9)	17.0783(4)	11.9186(3)
c(Å)	9.814(3)	9.8317(5)	6.8312(12)	12.7191(15)	20.3077(11)	5.9558(3)
α(deg)	90	90	90	96.093(3)	90	90
β(deg)	105.271(7)	90.281(2)	127.524(5)	98.513(3)	90	90
γ(deg)	90	90	90	101.750(3)	120	120
$V(A^3)$	695.3(3)	1170.47(10)	1305.3(4)	593.64(12)	5129.6(4)	732.69(5)
Z	4	2	4	2	6	10
T(K)	298(2)	298(2)	298(2)	298(2)	298(2)	298(2)
λ(Å)	0.71073	0.71073	0.71073	0.71073	0.71073	0.71073
Maximum 2θ(deg)	27.573	27.548	25.997	26.000	24.991	32.095
ρ calcd (g cm^{-3})	3.302	2.704	2.534	2.706	3.386	4.573
μ(Mo Kα)	6.538	4.746	4.596	5.379	11.621	33.355
R_1	0.0111	0.0322	0.0318	0.0227	0.0364	0.2004
wR_2	0.0286	0.0706	0.0726	0.0598	0.0610	0.5738

* The crystals of LuBOCl-6 have severe twinning issue and the precise atomic coordinates are therefore not available. Only the unit cell parameter information is provided while other physical parameters are given in the table based on an incomplete crystallographic solution.
